# Modelling the effects of antibiotic usage in livestock on human salmonellosis

**DOI:** 10.1016/j.onehlt.2023.100639

**Published:** 2023-10-07

**Authors:** Alex L.K. Morgan, Mark E.J. Woolhouse, Jaap A. Wagenaar, Bram A.D. van Bunnik

**Affiliations:** a Centre for Immunity, Infection & Evolution and School of Biological Sciences, University of Edinburgh, Edinburgh, United Kingdom; b Department of Environmental Systems Science, ETH Zürich, Zürich, Switzerland; c Usher Institute, University of Edinburgh, Edinburgh, United Kingdom; d Division of Infectious Diseases and Immunology, Faculty of Veterinary Medicine, Utrecht University, Utrecht, Netherlands; e Wageningen Bioveterinary Research, Lelystad, Netherlands; f WHO Collaborating Center for Reference and Research on Campylobacter and Antimicrobial Resistance from a One Health Perspective/WOAH Reference Laboratory for Campylobacteriosis, Utrecht, Netherlands; g Roslin Institute, University of Edinburgh, Midlothian, United Kingdom

**Keywords:** Antimicrobial resistance, Foodborne disease, Mathematical model, Antibiotic reduction, One health

## Abstract

Antibiotic usage in livestock has been suggested as a driver of antimicrobial resistance in human and livestock populations. This has contributed to the implementation of stewardship programs to curtail usage of antibiotics in livestock. However, the consequences of antibiotic curtailment in livestock on human health are poorly understood. There is the potential for increases in the carriage of pathogens such as *Salmonella* spp. in livestock, and subsequent increases in human foodborne disease. We use a mathematical model fitted to four case studies, ampicillin and tetracycline usage in fattening pig and broiler poultry populations, to explore the impact of curtailing antibiotic usage in livestock on salmonellosis in humans.

Increases in the daily incidence of salmonellosis and a decrease in the proportion of resistant salmonellosis were identified following curtailment of antibiotic usage in livestock. The extent of these increases in human foodborne disease ranged from negligible, to controllable through interventions to target the farm-to-fork pathway. This study provides a motivating example of one plausible scenario following curtailment of antibiotic usage in livestock and suggests that a focus on ensuring good farm-to-fork hygiene and livestock biosecurity is sufficient to mitigate the negative human health consequences of antibiotic stewardship in livestock populations.

## Highlights

• Curtailment of antibiotic usage in livestock can potentially increase the incidence of salmonellosis in humans.
• The extent of these increases in salmonellosis are negligible to controllable with interventions.
• Interventions targeting the farm-to-fork pathway are effective at controlling increases in incidence.

## 1 Introduction

A growing number of key antibiotic therapeutics are being rendered ineffective by antimicrobial resistance (AMR). Antibiotic usage in livestock has been identified as an important driver of AMR in human populations, with transmission of resistant bacteria and resistance determinants potentially occurring at the livestock/human interface [1]. This has led to efforts to curtail the usage of antibiotics in livestock [2,3]. The aims of these curtailment strategies are to safeguard the efficacy of clinical antibiotics and reduce the potential for transmission of resistant pathogens to human populations.

Curtailment of antibiotic usage in livestock has often resulted in desired reductions to AMR, with an example being reductions to faecal *Enterococci* resistance rates following EU growth promotion bans [[4], [5], [6]]. However, these reductions in usage have also been associated with transient increases in the carriage of other resistant pathogens, increases in livestock carriage of foodborne pathogens and increases in therapeutic antibiotic usage in livestock [[7], [8], [9]]. However, arguments have been made that these negative consequences can be largely attributed to increases in livestock productivity [[10], [11], [12]].

The uncertainty surrounding the consequences of curtailing antibiotic usage in livestock highlights the risks of introducing interventions into highly complex and poorly understood population/microbial level systems that have been built up through decades of antibiotic use as part of a “precautionary principle” based approach [9]. The need to better understand the potential long-term impacts of future AMR policy is also likely to increase, with EU legislation strictly controlling the use of antibiotics in livestock for metaphylaxis or prophylaxis in 2022 [3]. Therefore, there is a need for an increased understanding into the potential human health consequences following curtailment of antibiotics in livestock, especially when placed into a “one health” context.

A deterministic mathematical model was developed to explore the effects of antibiotic curtailment in livestock on *Salmonella* spp. infections in humans. Salmonellosis was explicitly chosen as a case study due to the clear zoonotic link between livestock carriage of *Salmonella* spp. and human infections. We explore the potential long-term consequences of antibiotic curtailment in livestock, including alterations to the overall incidence of human salmonellosis and the antibiotic-resistant fraction of infections. Additionally, we explore the effects and feasibility of introducing interventions to mitigate the potential negative consequences of antibiotic curtailment in livestock.

## 2 Methodology

### 2.1 Model structure and description

**Fig. 1:**
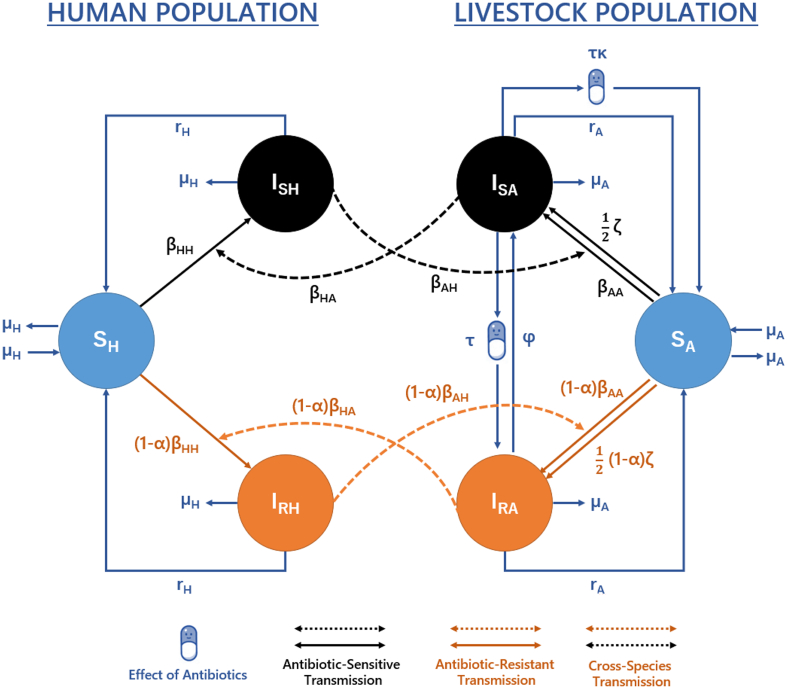
Model structure describing the transmission of foodborne pathogens between/within livestock and human populations. Model equations and parameters can be found described in the *Supplementary Material* (*eq. S1.1*, *Table S5*).

Each host population can be stratified based on their respective infection status: susceptible humans (S_H_), humans infected with antibiotic-sensitive (I_SH_) or antibiotic-resistant *Salmonella* spp. (I_RH_), susceptible livestock (S_A_) and livestock infected with antibiotic-sensitive (I_SA_) or antibiotic-resistant *Salmonella* spp. (I_RA_) (Fig. 1). For simplicity, we considered “infected” states in livestock to also include asymptomatic carriage. Transmission is simplified into four transmission routes: animal-to-animal (β_AA_), human-to-human (β_HH_), animal-to-human (β_HA_) and human-to-animal (β_AH_) transmission.

A background transmission rate from the environment or new introductions from other (non) considered populations to the livestock population was also modelled (ζ). This was scaled by a factor of 0.5 to ensure an equal influence on sensitive and resistant transmission, with this value chosen due to a lack of a priori information on differences in background contamination rates between sensitive and resistant strains. Natural recovery from antibiotic-sensitive/resistant infection occurs in both human/livestock populations at rate r_H_ and r_A_ respectively. Per capita birth/death rates are represented by μ_A_ in livestock and μ_H_ in human populations.

Antibiotic usage was modelled as a rate (τ) and was assumed to have a combined therapeutic and selective pressure on antibiotic-sensitive *Salmonella* spp. infection. This therapeutic effect was assumed to both shorten the duration of carriage and clear antibiotic-sensitive infection. Due to the unclear relationship between antibiotic usage and clearance of *Salmonella* spp. in livestock species, a scaling parameter was also included to describe the efficacy of antibiotic mediated recovery in livestock (κ). The selective pressure of antibiotics was modelled to convert livestock from antibiotic-sensitive to resistant states. This could be interpreted as a majority-minority relationship, with antibiotic usage clearing sensitive bacteria, allowing an implicitly modelled minority antibiotic-resistant (I_RA_) strain to proliferate and dominate, leading to “conversion” [13].

A reversion rate (φ) was also used to encompass a range of different mechanisms that may cause reversion of antibiotic-resistant (I_RA_) to sensitive (I_SA_) infection. For example, this rate may describe within-host growth-mediated competition, where sensitive strains may outcompete resistant strains in the absence of antibiotics. The absence of antibiotics is captured through the antibiotic treatment rate (τ), with this rate implicitly assuming that while some livestock are treated and exposed to antibiotics, others may not be.

Transmission-related fitness costs associated with antibiotic-resistance were assumed to reduce the rate of transmission (α). This can be interpreted as a decrease in capacity for resistant strains (relative to sensitive strains) to establish infectious carriage due to changes in important cellular machinery needed to facilitate resistance to antibiotics [[14], [15], [16]].

### 2.2 Primary outcome measures

Two primary outcome measures were considered in this study: 1) the daily incidence of human non-typhoidal human salmonellosis per 100,000 population in the EU. Details on this incidence calculation can be found in the S*upplementary Material*. 2) The fraction of antibiotic-resistant human non-typhoidal salmonellosis (I^⁎^_RHProp_) (defined as I_RH_ / (I_SH_ + I_RH_)). Both measures were calculated at the long-term non-zero steady state.

Studying disease dynamics at equilibrium is a useful indication of where the modelled system is heading. This is especially the case for resistant *Salmonella* spp. infections, with a short duration of infectious human carriage (1/r_H_), facilitating a rapid approach to equilibrium and with temporal surveillance data suggesting a recent plateau in the proportion of antibiotic resistance in livestock populations (*Fig. S1–4*).

### 2.3 Case studies and datasets

To accurately describe the relationship between antibiotic usage in livestock and resistance, the model was fitted using an approximate Bayesian computation sequential Monte-Carlo (ABC-SMC) using resistance/sales surveillance data. Detailed methodology for the ABC-SMC approach can be found in Toni et al., (2009) [17].

The proportion of isolates resistant to the specific antibiotic class from carcasses of broiler poultry/fattening pigs was extracted from the respective European Food Safety Authority (EFSA) datasets [[18], [19], [20], [21], [22], [23]]. Antibiotic sales data was obtained from European Surveillance of Veterinary Consumption (ESVAC) reports [[24], [25], [26], [27], [28]]. Note that due to a lack of accurate country-level antibiotic usage data, sales were assumed to be a proxy for usage. Details of the raw datasets and data manipulation of the ESVAC and EFSA datasets can be found in the *Supplementary Material*.

Four case studies were chosen to aid model parameterisation. These case studies were: 1) ampicillin-resistant non-typhoidal salmonella in broilers from 2014 to 2018, 2) tetracycline-resistant non-typhoidal salmonella in broilers from 2014 to 2018, 3) ampicillin-resistant non-typhoidal salmonella in fattening pigs from 2015 to 2018 and 4) tetracycline-resistant non-typhoidal salmonella in fattening pigs from 2015 to 2018.

These four case studies were chosen due to the high level of usage (both historical and current) of tetracycline and ampicillin in broilers and fattening pigs, and the availability of resistance data for these two livestock species [[24], [25], [26], [27], [28], [29]]. A statistically significant relationship between usage and resistance was identified for three out of four included case studies (*Fig. S5*, *Table S2*).

### 2.4 ABC-SMC model fitting procedure

A sum of squared errors distance function was used to calculate the distance between the simulated and observed fraction of antibiotic-resistant livestock infection for each country/year data point in the ABC-SMC inference process [18,19,22,23].

Two additional summary statistics were also used for ABC-SMC model fitting: 1) minimise the difference between the modelled and observed daily EU incidence of human salmonellosis currently observed (0.593 per 100,000) at baseline antibiotic usage, 2) minimise the difference between the modelled and observed proportion of resistant human salmonellosis for each case study at baseline antibiotic usage. Note that this baseline EU incidence of human salmonellosis was obtained from the European Surveillance System (TESSy) reports and converted from a prevalence value [30]. Details can be found in the Supplementary Material [30].

The baseline antibiotic usage for each case study was considered the unweighted average tetracycline/ampicillin usage across each antibiotic country/year data point. 1) Ampicillin-resistant *Salmonella* spp. in broiler poultry (0.314 at 0.0049 g/PCU), 2) tetracycline-resistant *Salmonella* spp. in broiler poultry (0.316 at 0.0069 g/PCU), 3) ampicillin-resistant *Salmonella* spp. in fattening pigs (0.345 at 0.0125 g/PCU) and 4) tetracycline-resistant *Salmonella* spp. in fattening pigs (0.340 at 0.01305 g/PCU).

### 2.5 Fitted parameters

The ABC-SMC approach was used to estimate the marginal posterior probability distribution for six model parameters$,\theta =\left[\beta_{\mathrm{AA}},\kappa ,\varphi ,\alpha ,\beta_{\mathrm{HA}},\zeta \right]$ [17]. Other model parameters were not fitted as estimates with high levels of certainty were available (r_H_, r_A_, μ_A_ and μ_H_), or due to the relative nature of other transmission parameters with respect to β_AA_, β_HA_ and ζ (β_HH_ and β_AH_). β_HH_ and β_AH_ were instead held at values of 0.0001. These values were chosen due to the negligible impact of these transmission routes on *Salmonella* spp. transmission [31]. Prior distributions and fitted model values can be found in the *Supplementary Material*.

### 2.6 Sensitivity analyses

A Fourier amplitude sensitivity test (FAST) approach was used to identify the impact of the model parameters on two direct-model outputs and two intervention-related model outputs [32]: 1) the daily incidence of human foodborne infection, 2) proportion of resistant human infection, 3) relative changes in daily incidence when antibiotic usage in livestock are curtailed (*τ* = 0 g/PCU), compared to daily incidence at mean baseline antibiotic usage across the four case studies (*τ* = 0.00934 g/PCU) and 4) relative changes in daily incidence under antibiotic curtailment (0 g/PCU) relative to the observed daily incidence with current levels of antibiotic usage (0.593 per 100,000). An in-depth description of this sensitivity analysis can be found in the *Supplementary Material*.

## 3 Results

**Fig. 2:**
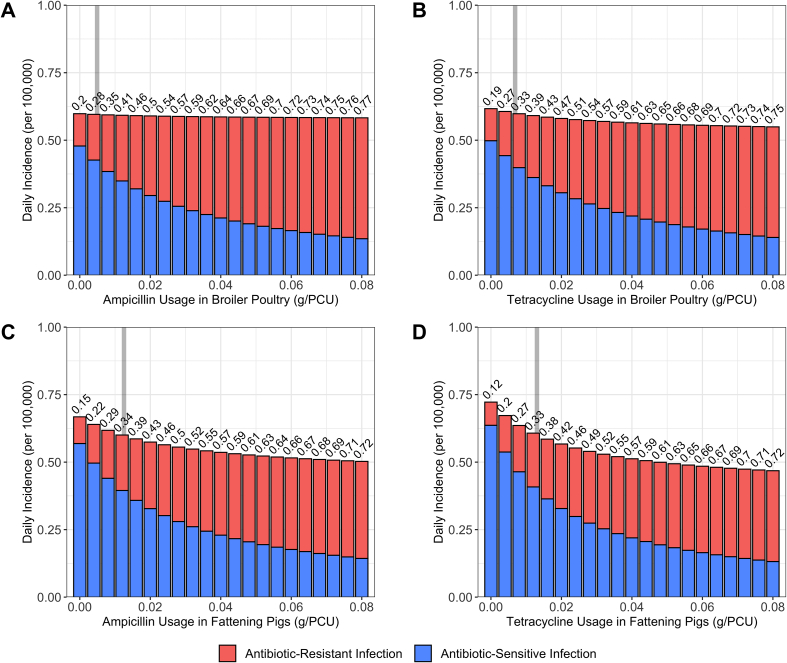
Impact of alterations in antibiotic usage in livestock (τ) on the daily incidence of human salmonellosis and the proportion of resistant human infection (I^⁎^_RHProp_). A) Ampicillin-resistant human salmonellosis from broiler poultry. B) Tetracycline-resistant human salmonellosis from broiler poultry. C) Ampicillin-resistant human salmonellosis from fattening pigs. D) Tetracycline-resistant human salmonellosis from fattening pigs. Grey bar denotes the case study specific baseline antibiotic usage in livestock (τ = 0.0035/0.0049/0.0081/0.0109). Numbers above the bars denote I^⁎^_RHProp_. Information on the model fitting procedure and the fitted daily incidence and I^⁎^_RHProp_ for each case study can be found in the *Supplementary Material* (Table S6).

Curtailment of antibiotic usage (τ → 0 g/PCU) in the fattening pigs case studies resulted in the largest increase in the daily incidence of human salmonellosis with a 1.11-fold (0.668 per 100,000) increase relative to baseline levels, and a 1.20-fold (0.720 per 100,000) for the ampicillin and tetracycline case studies respectively (Fig. 2) [30]. In contrast, increases in daily incidence for the broiler poultry case studies ranged from a zero-fold change below 3 significant figs. (0.598 per 100,000) for the ampicillin case study and a 1.02-fold (0.617 per 100,000) increase in daily incidence for the tetracycline usage case study.

**Fig. 3:**
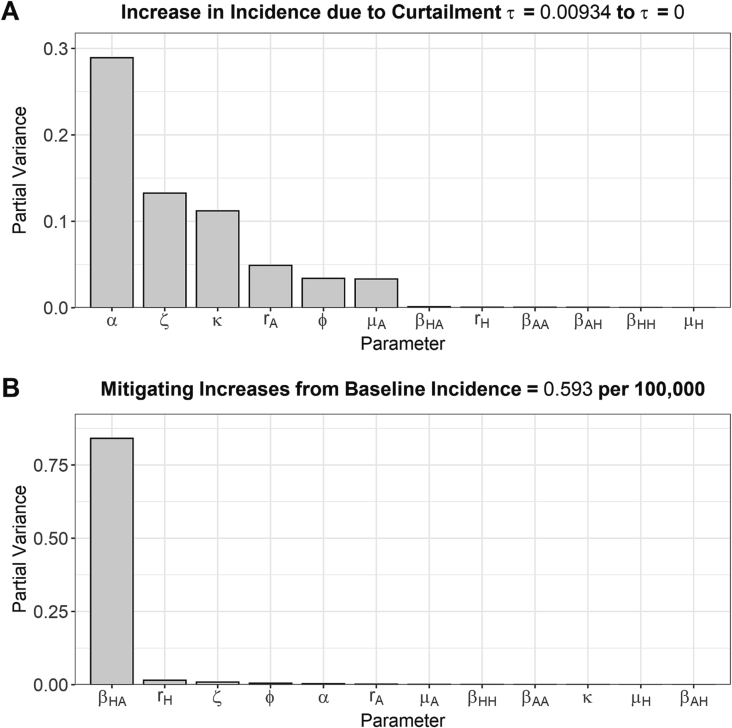
Fourier amplitude sensitivity test (FAST) to identify the most influential model parameter for: A) Relative change in daily incidence of human salmonellosis under curtailment (0 g/PCU) compared to the averaged baseline antibiotic usage level (0.00934 g/PCU). B) Mitigating changes in daily incidence of human salmonellosis under curtailment compared to the level of foodborne disease experienced under current levels of antibiotic usage in livestock (0.593 per 100,000 population). Higher bars indicate greater sensitivity. A FAST analysis of baseline model outcome measure, daily incidence and I^⁎^_RHProp_ was also performed (*Fig. S14*).

A Fourier amplitude sensitivity test (FAST) was next performed to identify the parameters which had the greatest influence on the relative increase in the daily incidence of human salmonellosis when antibiotic usage in livestock was curtailed from mean baseline usage (0.00934 → 0 g/PCU) (Fig. 3A). The FAST approach generates parameter combinations resulting in a different daily incidence at baseline antibiotic usage for each combination (τ = 0.00934 g/PCU). This can be interpreted as exploring case studies and scenarios other than the specific drug/livestock/pathogen combinations used as baseline scenarios in this study.

Transmission related fitness costs associated with antibiotic-resistance (α), the per capita rate of background transmission to livestock populations (ζ) and efficacy of antibiotic-mediated livestock recovery (κ) were found to be the most influential parameters in determining the relative increase in the daily incidence of human salmonellosis from baseline antibiotic usage in livestock when antibiotics where curtailed (Fig. 3A). Specifically, lower κ and α, and higher ζ parameter values resulted in lower relative increases in daily incidence when antibiotic usage in livestock was curtailed (τ → 0 g/PCU) (*Fig. S16*).

A follow up sensitivity analysis was performed to identify parameters that could best *mitigate* increases in the daily incidence of human salmonellosis under antibiotic curtailment to a value below 0.593 per 100,000 population, the incidence currently observed for the modelled case studies (Fig. 3B). The per capita rate of animal-to-human transmission (β_HA_) was identified as the key parameter to mitigate increases in daily incidence (Fig. 3B). This therefore represents the best parameter to target to mitigate potential increases in daily incidence due to curtailment of antibiotic usage in livestock.

Due to the importance of targeting the animal-to-human transmission route, we quantified the minimum alterations in β_HA_ required to prevent increases in daily incidence under antibiotic usage curtailment (τ → 0 g/PCU), above what is currently observed for human salmonellosis (0.593 per 100,000). Alterations to β_AA_ and ζ parameters were also chosen as potential intervention targets, due to their relevance in agricultural biosecurity strategies to mitigate livestock disease/AMR [33,34].

**Fig. 4:**
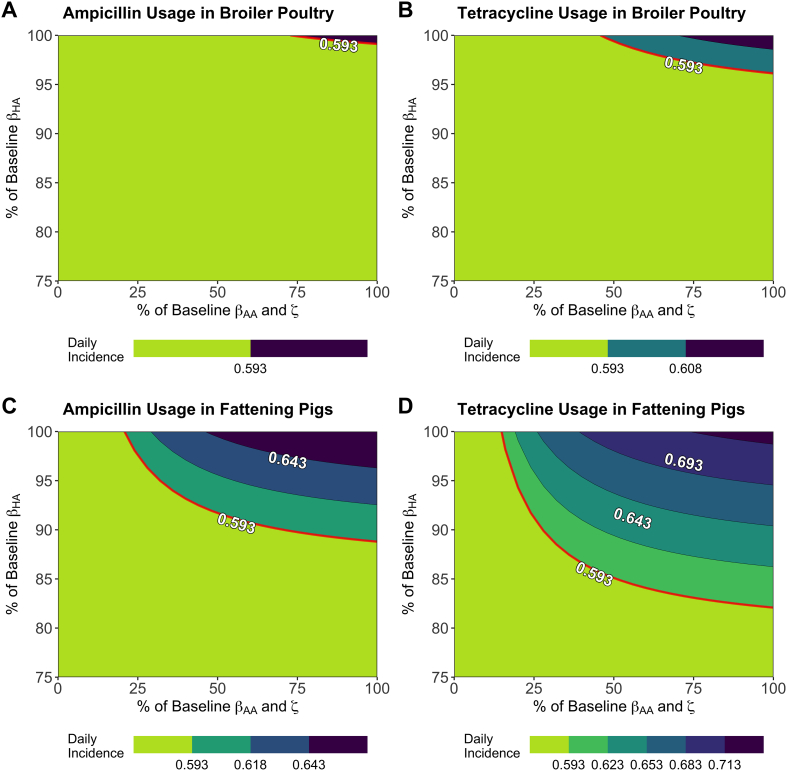
Reductions to key model parameters, animal-to-human transmission (β_HA_), animal-to-animal transmission (β_AA_) and the background transmission rate to animal populations (ζ) to mitigate increases in the daily incidence of human salmonellosis under curtailment of antibiotic usage in livestock (τ → 0 g/PCU). A) Ampicillin-resistance in broiler poultry, B) tetracycline-resistance in broiler poultry, C) ampicillin-resistance in fattening pigs and D) tetracycline-resistance in fattening pigs. Axes represent interventions that reduce the labelled transmission rate(s) to % of their original values. Note that the top right corner of each contour plot represents a scenario with curtailment of antibiotics and no further alterations to any model parameter. The red line represents the threshold at which daily incidence is below current levels (0.593 per 100,000). Note the asymmetrical % reduction for both x and y-axis. (For interpretation of the references to colour in this figure legend, the reader is referred to the web version of this article.)

Only reductions to β_HA_ were capable of mitigating increases to the daily incidence of human salmonellosis below baseline levels across all case studies in the explored parameter space, with a reduction of 1%, 4%, 12% and 18% required for each case study (Fig. 4). Isolated or even combined reductions to β_AA_ or ζ were only capable of reducing daily incidence below baseline levels with strong reductions below ∼50%, or if the initial increase in daily incidence was negligible upon antibiotic curtailment, as seen with the ampicillin usage in broiler poultry case study (Fig. 4A).

## 4 Discussion

A mathematical modelling approach was used to identify increases in the daily incidence of non-typhoidal human salmonellosis following curtailment of antibiotic usage in livestock. This was explored across four antibiotic/livestock specific case studies. Scenarios with high transmission-related fitness costs of resistance (α), high efficacies of antibiotic-mediated livestock recovery (κ) and low background transmission rates of *Salmonella* spp. in livestock (ζ) were found to result in large increases in the daily incidence of human salmonellosis upon antibiotic curtailment. However, interventions to decrease animal-to-human transmission (β_HA_) were found to effectively mitigate increases in the daily incidence of human salmonellosis following curtailment of antibiotic usage in livestock.

Reductions to β_HA_ could take the form of interventions to increase hygiene throughout the farm-to-fork pathway, reducing microbial contamination on carcasses, as well as public information campaigns to promote safe handling of food products [33,35]. Many of these interventions have already been implemented which could be a promising signal that current business-as-usual approaches could be sufficient to control increases in foodborne disease following future antibiotic usage stewardship [[36], [37], [38]]. However, *Salmonella* spp. incidence has also plateaued in regions [39]. There will also likely be large heterogeneity in the impact of different interventions to improve hygiene at the farm-to-fork pathway to reduce β_HA_ [40]. This may be an indication that further reductions to incidence, if not already reduced by current interventions to reduce transmission, may be difficult to achieve.

Curtailment of antibiotic usage in livestock was found to have varying impacts across the modelled livestock host species. This can be attributed to the differences in transmission-related fitness costs associated with antibiotic resistance between species (α = 0.084 and 0.416 for broiler poultry and fattening pigs respectively). Difference in fitness cost between species may reflect heterogeneity in the distribution of *Salmonella* spp. serotypes colonising poultry and pig hosts [41]. Heterogeneity in fitness cost across hosts could also be attributable to distinct plasmid types in chickens and pigs, with studies in *E.coli* identifying differences in fitness cost across these resistance-encoding plasmids [42].

In addition to α, differences in the relative increase in daily incidence of human salmonellosis between modelled case studies can be attributed to ζ and κ parameters (Fig. 3A). The effects of changes in these parameters on the impact of curtailment are twofold: Firstly, treatments which have a greater therapeutic impact on the duration of antibiotic-sensitive carriage, $\left(\frac{1}{\tau \kappa +r_{A}}\right)$, will intuitively result in larger increases in prevalence when withdrawn (high κ) (*Fig. S17*). Secondly, greater transmission-related fitness costs (high α) and import of sensitive bacteria from the environment (high ζ) will promote a greater relative proportion of sensitive to resistant strains (Fig. S18). Therefore, when sensitive strains are more common, we will observe a greater increase in incidence of human salmonellosis when treatment is withdrawn, as sensitive strains are the only strain affected by antibiotic pressure.

Antibiotic usage in livestock was also modelled to be a proxy for all modes of application (meta-phylaxis, prophylaxis etc.) and therefore by extension, our model implicitly assumes that all types of antibiotic usage have a therapeutic effect in livestock. This assumption can be considered an edge-case, highly positive interpretation of antibiotic usage in livestock, considering that the impact of antibiotic exposure to *Salmonella* spp. carriage in livestock is highly variable and antibiotic dependent [43,44]. However, the fact that increases in human incidence are still minor under an optimistic assumption that curtailment is occurring to antibiotic usage with a highly therapeutic effect in livestock, further reinforces the message that the real-life impact of antibiotic curtailment on salmonellosis will likely be minimal.

It is also likely that there will be a less clear link between improvements in farm-to-fork hygiene and the incidence of opportunistic infections of commensal pathogens, such as *Listeria* spp. and *E.coli* (i.e. VTEC). This contrasts with *Salmonella* spp. modelled in this study, which has clear food animal origins and predominantly results in self-limited colonisation and infection in humans [31]. Factors such as the extent of host-immunosuppression, microbial community interactions and nosocomial transmission may play a larger role than the animal-to-human transmission pathway in determining the extent of *Listeria* spp. and *E.coli* infection in humans [45,46].

Due to the historical lack of high-quality AMR surveillance and presence of confounding factors, it is difficult to disentangle whether observed significant relationships between usage and resistance are due to a genuine relationship between usage and resistance or due to noise associated with surveillance data (*Fig. S5*, *Table S2*) [47]. However, our key message, specifically that increases in the daily incidence are likely to be low and controllable through interventions, is robust to these uncertainties and variations in the data. For example, if the true relationship between usage and resistance was not significant, then we would expect to see negligible increases in the daily incidence of foodborne disease in humans. This is because of transmission-related fitness costs (α) being an important parameter in driving changes in resistance and increases in the incidence upon curtailment (Fig. 4A, *S17*). Therefore, if there is a weak/no association between antibiotic usage and resistance due to negligible fitness costs, then increases in incidence will also be unimportant and of limited public health concern.

The compartmental model structure chosen in this study was simplified for model tractability and certain phenomena were implicitly assumed or modelled. For example, transmission of *Salmonella* spp. from animals-to-humans was simplified using a single parameter in this study. Future models could use non-linear microbial load dose-response models to more accurately quantify infection risk upon human exposure to *Salmonella* spp. on food products [48]. Future models could also explicitly model mechanisms driving strain coexistence, such as within-host competition, with this mechanism known to impact AMR dynamics following the implementation of interventions [49]. The impact of country level adherence to antibiotic curtailment interventions on human and livestock AMR could also be of interest, to explore the impact of population structure on intervention efficacy. Finally, the relationship between livestock antibiotic usage and resistance was assumed to be linear in this study, with this also being assumed in related literature [49,50]. Exploring the functional form of this relationship may also provide useful insight into the range of potential scenarios following curtailment interventions at the one health interface.

The results from this study suggest that curtailment of antibiotic usage in livestock may have unforeseen effects, with a reduction in both livestock and human antibiotic resistance, but with increases in the livestock carriage and onwards transmission of foodborne pathogens such as *Salmonella* spp. to humans. However, potential increases in the daily incidence of salmonellosis range from negligible to preventable through interventions that target animal-to-human transmission routes. The efficacy of these interventions suggests that a one-health approach with a focus on improving farm-to-fork hygiene to minimise human disease is essential when considering potential strategies to tackle the AMR crisis.

## Authors' contributions

A.L.K.M. participated in the study design, carried out model analysis and drafted the manuscript. B.A.D.v.B. participated in the study design and provided feedback on manuscript drafts. M.E.J.W. participated in the study design and provided feedback on manuscript drafts. J.A.W. provided feedback on manuscript drafts.

## Funding

This study was supported by a 10.13039/100010269Wellcome Trust PhD grant (215094/Z/18/Z), 10.13039/501100004191Novo Nordisk (684/21856), 10.13039/501100000780European Union VEO grant (874735-VEO), the 10.13039/501100000848University of Edinburgh and 10.13039/501100003006ETH Zürich.

## Declaration of Competing Interest

All authors declare that they have no conflicts of interest.

## Data Availability

Datasets and reproducible code can be found available from https://github.com/alexmorgan1995/FoodborneDisease.

## Appendix A Supplementary data

Supplementary material (text and figures) Image 1
