# Supplementary material for: Modelling the effects of antibiotic usage in livestock on human salmonellosis
Source: One Health. 2023 Oct 7;17:100639. doi: 10.1016/j.onehlt.2023.100639 (PMC10665166; doi:10.1016/j.onehlt.2023.100639)
Supplement: Supplementary file 1 — Supplementary material (text and figures) [file mmc1.docx]

***SUPPLEMENTARY MATERIAL -*** *Modelling the effects of antibiotic usage in livestock on human salmonellosis*

**Model Equations**

$$\frac{dS_{A}}{dt}=r_{A}\left( I_{SA}+I_{RA} \right)+\tau\kappa I_{SA}-\beta_{AA}S_{A}\left[ I_{SA}+I_{RA}\left( 1-\alpha\right) \right]-\beta_{AH}S_{A}\left[ I_{SH}+I_{RH}\left( 1-\alpha\right) \right]-\frac{1}{2}\zeta S_{A}-\frac{1}{2}\zeta S_{A}\left( 1-\alpha\right)-\mu_{A}\left( S_{A}-1 \right)$$

$$\frac{dI_{SA}}{dt}=\beta_{AA}I_{SA}S_{A}+\beta_{AH}I_{SH}S_{A}+\varphi I_{RA}+\frac{1}{2}\zeta S_{A}-I_{SA}\left( \mu_{A}+r_{A}+\tau+\tau\kappa\right)$$

$$\frac{dI_{RA}}{dt}=\beta_{AA}I_{RA}S_{A}\left( 1-\alpha\right)+\beta_{AH}I_{RH}S_{A}\left( 1-\alpha\right)+\tau I_{SA}+\frac{1}{2}\zeta S_{A}\left( 1-\alpha\right)-I_{RA}\left( \mu_{A}+r_{A}+\varphi\right)$$

$$\frac{dS_{H}}{dt}=r_{H}\left( I_{SH}+I_{RH} \right)-\beta_{HH}S_{H}\left[ I_{SH}+I_{RH}\left( 1-\alpha\right) \right]-\beta_{HA}S_{H}\left[ I_{SA}+I_{RA}\left( 1-\alpha\right) \right]-\mu_{H}\left( S_{H}-1 \right)$$

$$\frac{dI_{SH}}{dt}=\beta_{HH}I_{SH}S_{H}+\beta_{HA}I_{SA}S_{A}-I_{SH}\left( \mu_{H}+r_{H} \right)$$

$$\frac{dI_{RH}}{dt}=\beta_{HH}I_{RH}S_{H}\left( 1-\alpha\right)+\beta_{HA}I_{RA}S_{A}\left( 1-\alpha\right)-I_{RH}\left( \mu_{H}+r_{H} \right)$$

Eqn S1.1

**Datasets**

Yearly ESVAC data for livestock antibiotic sales and EFSA data on the proportion of *Salmonella* spp. isolates obtained from livestock species carcasses resistant to the tetracycline/ampicillin were used to create usage/sales and livestock resistance pairs for each country in each respective case study [1-11]. These pairs spanned across multiple years for each country (Figure S1-4). Therefore, for any one country, there may be multiple usage/resistance pairs corresponding to different years in the dataset. These pairs were used to determine the observed relationship between livestock antibiotic sales/usage and the fraction of antibiotic-resistant livestock infection in European countries between 2014-2018 for each respective livestock species.


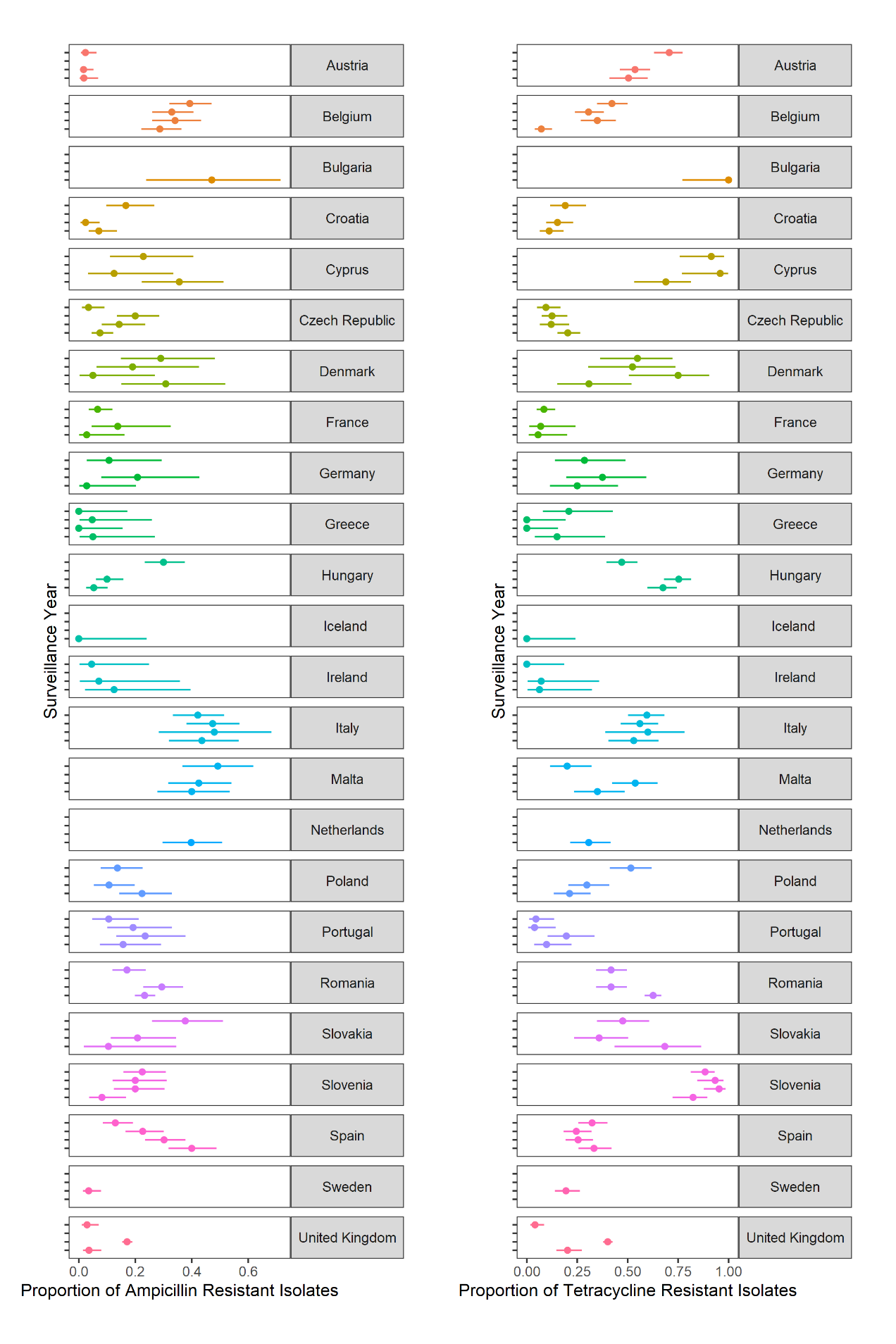


**Figure S1. Stability of ampicillin and tetracycline resistance during 2014-2018 across each country in the EFSA data set for broiler poultry.** Note that the unmarked Y-axis corresponds to 2014 (lowest) to 2018 (highest) for all countries.


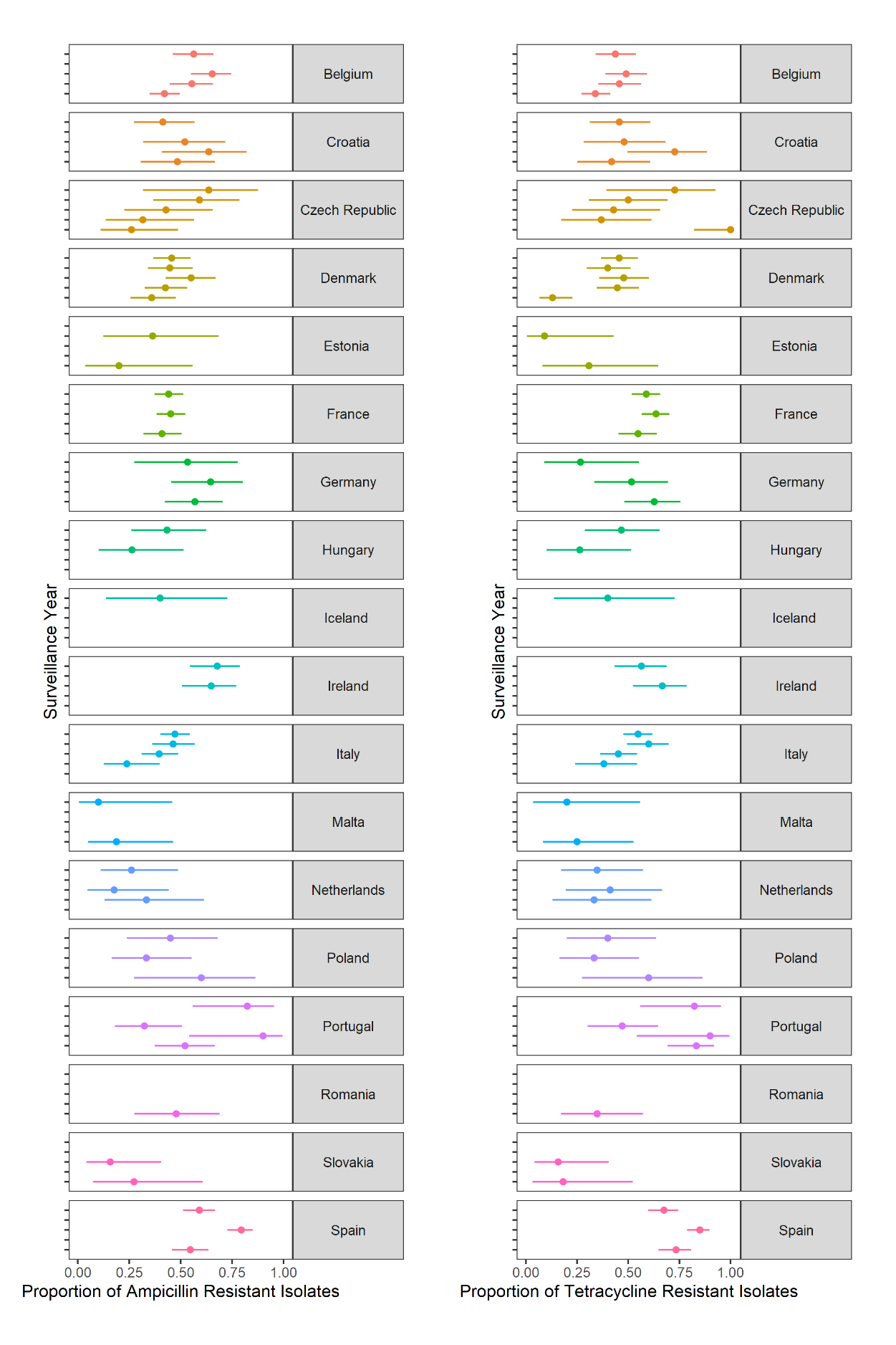


**Figure S2. Stability of ampicillin and tetracycline resistance during 2014-2019 across each country in the EFSA data set for fattening pigs.** Note that the unmarked Y-axis corresponds to 2014 (lowest) to 2019 (highest) for all countries.


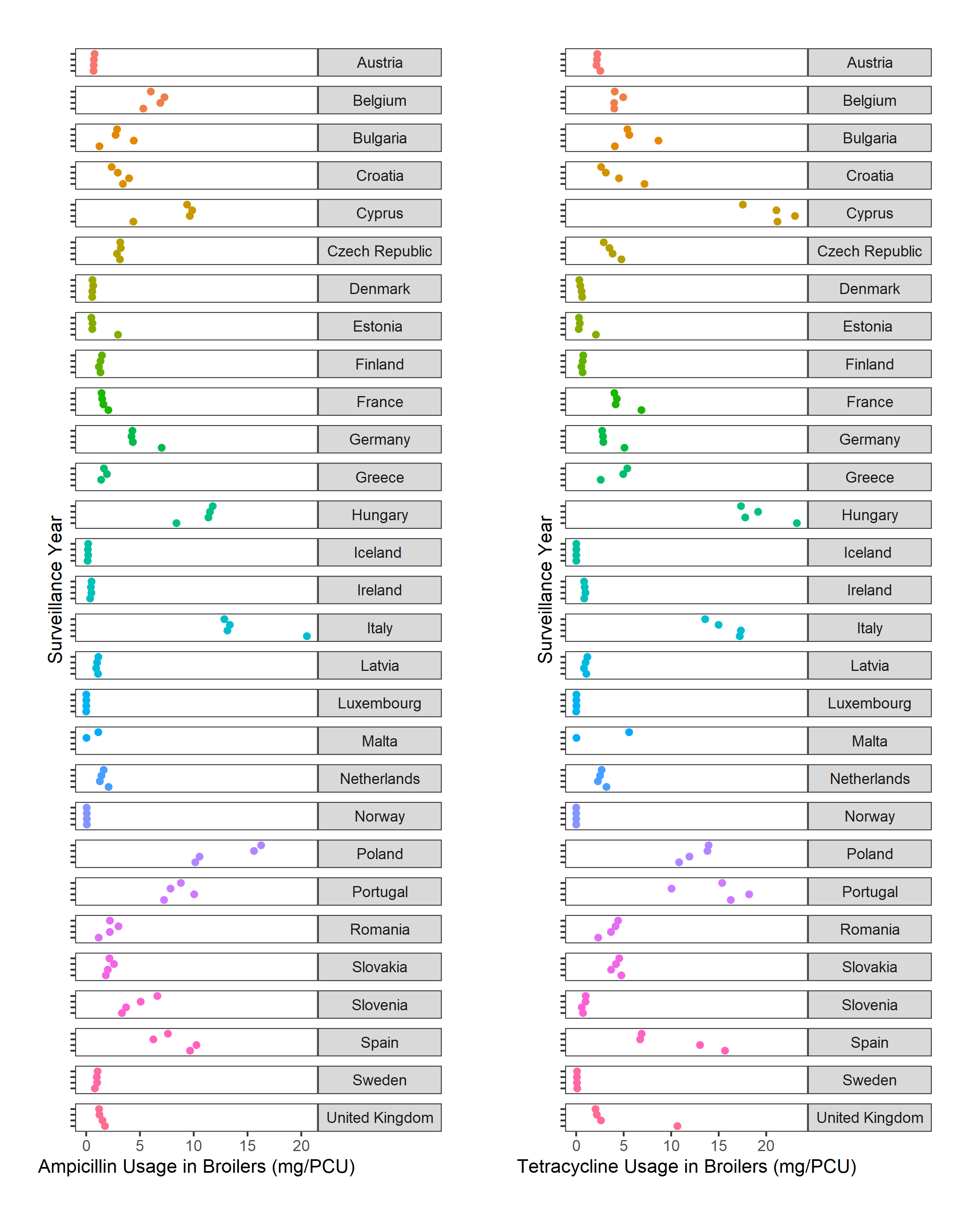


**Figure S3. Stability of ampicillin and tetracycline usage during 2014-2018 across each country in the ESVAC data set for broiler poultry.** Note that the unmarked Y-axis corresponds to 2014 (lowest) to 2018 (highest) for all countries.


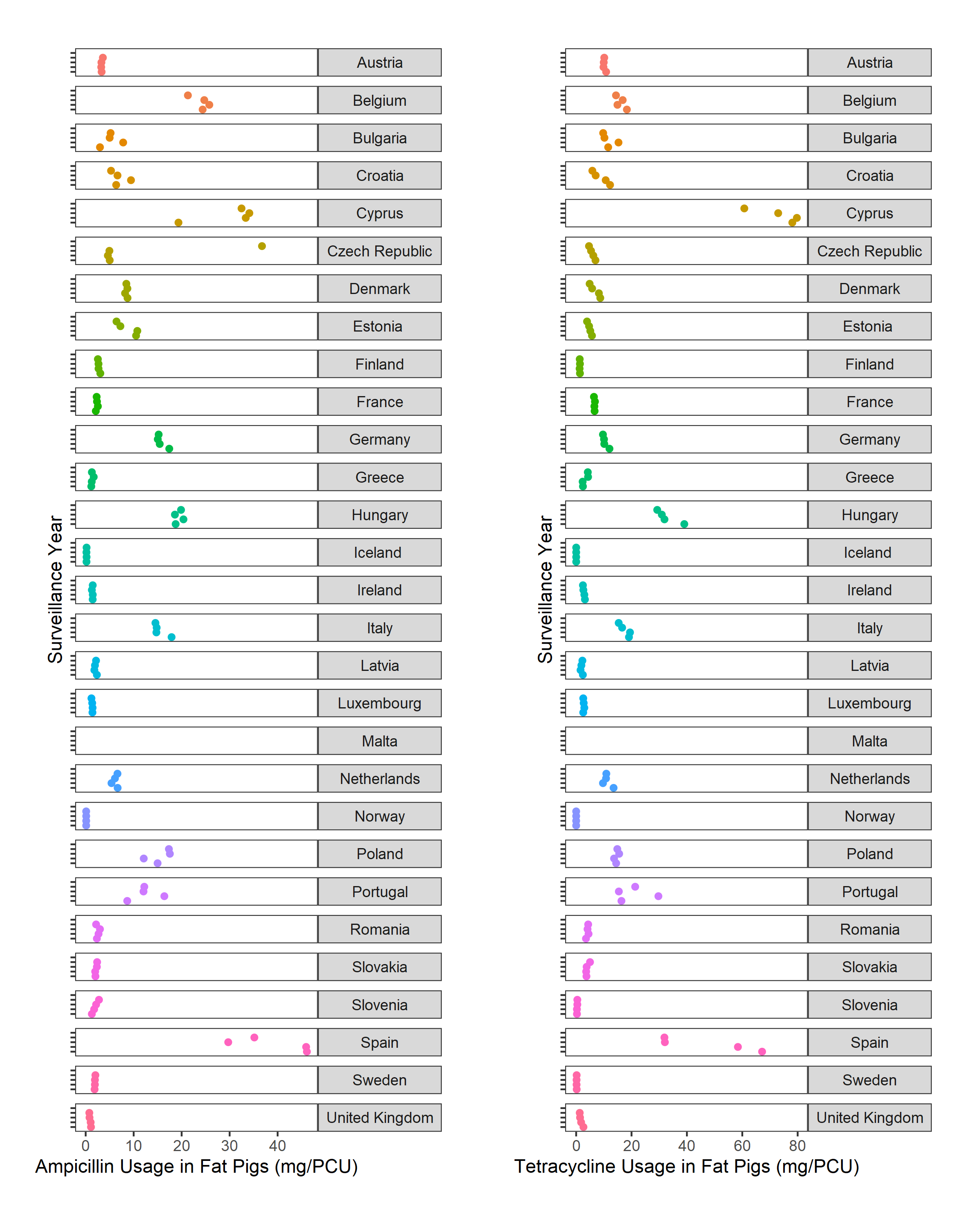


**Figure S4. Stability of ampicillin and tetracycline usage during 2014-2019 across each country in the ESVAC data set for fattening pigs.** Note that the unmarked Y-axis corresponds to 2014 (lowest) to 2019 (highest) for all countries.

It is important to note that the stratification of each country into their respective yearly data for each data point introduces an assumption that the level of antibiotic usage will also be representative of resistance for a particular year. Due to the existence of lag between the effects of antibiotic stewardship interventions and alterations in either human or livestock resistance [12], it is important to ensure that there are relative levels of stability in the yearly usage and resistance for each country. We note that for the majority of included countries, this temporal stability for each country across included yearly data points was observed, therefore we can be confident that resistance will still correspond to usage in the explored time period.

**ESVAC Antibiotic Sales Data Scaling**

European Surveillance of Veterinary Antimicrobial Consumption (ESVAC) antibiotic sales data was used in this study as a proxy for livestock antibiotic usage [1-5]. This measure was used due to a lack of livestock species-specific antibiotic usage surveillance data stratified by country. We note that livestock antibiotic sales are not an exact proxy measure for usage, nor has a definitive link been proven between these two quantities. However, due to a lack of more relevant epidemiological surveillance data, the ESVAC dataset was deemed sufficient.

The ESVAC dataset provides antibiotic sales expressed in mg/PCU for all livestock, representing a composite measure of milligram (mg) of active ingredient normalised by the population correction unit (PCU). This latter measure, PCU, can be considered the total biomass of all livestock populations potentially treatable with antimicrobials. A scaling calculation was conducted to scale the non-specific overall livestock antibiotic sales to be species-specific for each case study.

This scaling was performed by first identifying the proportion PCU of the particular livestock species of interest in each case study, in relation to the total livestock PCU in each country. This country-specific proportion was then used to scale the level of antibiotic usage (mg/PCU) for the specific livestock species of interest for each country. This was repeated for every included year for each country in each case study. Note that for this study, g/PCU was used as the antibiotic sales unit of measurement for all model fitting. An example of this scaling calculation using the average across each considered country in the dataset for each case study can be found below (countries with n > 10 samples) (Table S1).

**Table S1. Scaling for species-specific antibiotic sales using the averages across included countries/years as illustrative example.**

| Case Study | Livestock Species | Antibiotic Class | Total PCU | Livestock-specific PCU | Fraction of the country PCU | Country level Antibiotic Sales  (mg/PCU) | Scaled Antibiotic Sales  (mg/PCU) |
| --- | --- | --- | --- | --- | --- | --- | --- |
| **1 (n = 69)** | Broiler Poultry | Ampicillin | 2676.90 | 2134.717 | 0.142828 | 24.46256 | 3.493929 |
| **2 (n = 69)** | Broiler Poultry | Tetracycline | 2676.90 | 2134.717 | 0.142828 | 34.02772 | 4.860097 |
| **3 (n = 37)** | Fattening Pigs | Ampicillin | 2134.86 | 693.0439 | 0.324632 | 25.07895 | 8.14143 |
| **4 (n = 37)** | Fattening Pigs | Tetracycline | 2134.86 | 693.0439 | 0.324632 | 33.45789 | 10.8615 |

The final scaled measure is therefore the country-level antibiotic sales scaled by the livestock-specific biomass and not necessarily the livestock-specific level of antibiotic usage for each country. However, in lieu of more accurate antibiotic usage surveillance data, this proxy measure was used in the model fitting process.

**Community-level scaling for the overall prevalence of human salmonellosis**

Access to EU surveillance data for human non-typhoidal salmonellosis is available through The European Surveillance System (TESSy) annual epidemiological reports [13]. However, factors such as under-ascertainment (health seeking behaviour) and underreporting of non-typhoidal salmonellosis will likely result in the reported incidence being an underestimate of community-level transmission. Use of multiplication factors have been proposed, which uses a scaling factor to upscale incidence rates in surveillance reports to more accurately reflect community-level incidence [14]. We aimed to capture this community-level rather than the reported incidence of non-typhoidal salmonellosis for use in this study.

Data from the Burden of Communicable Disease in Europe (BCoDE) study was used to obtain an estimate for the average community incidence of non-typhoidal salmonellosis in EU/EEA countries [14]. The BCoDE study was undertaken from 2009-2013, occupying a different timeframe from the resistance data used in the ABC-SMC model fitting (2014-2018) [6-11]. However, due to a recent plateau in the absolute incidence of non-typhoidal salmonellosis over the last decade, we assumed that this BCoDE data could be extrapolated to the more recent timeframe of the resistance data [13]. This is barring any extensive European demographic changes over the last decade (denominator) which would alter the incidence per unit population.

From the BCoDE data, an annual community-level incidence of 216.46 per 100,000 was identified for non-typhoidal salmonellosis averaged across sex and age groups. To convert this estimate into a daily incidence usable in this study, this annual incidence was divided by 446,000,000 [15]. This resulted in a European community-level estimate for the daily incidence of 0.593 per 100,000 population. This was used as the model baseline for the overall daily incidence of human non-typhoidal salmonellosis in Europe under current livestock antibiotic usage levels.

**Relationship between antibiotic usage and resistance**

A linear regression was condcted as an exploratory analysis to investigate the presence of a relationship between antibiotic sales (as a proxy for usage) and antibiotic resistance for the four model case studies. Antibiotic usage was considered the explanatory variable, with model coefficents presented in Table S2.

**Table S2. Linear regression of model case study data.**

| Variable | Case Study | | | |
| --- | --- | --- | --- | --- |
|  | **Ampicillin Resistance in Broiler Poultry** | **Tetracycline Resistance in Broiler Poultry** | **Ampicillin Resistance in Fattening Pigs** | **Tetracycline Resistance in Fattening Pigs** |
| **Intercept Constant** | 0.1216 | 0.3120 | 0.3692 | 0.400879 |
| **Antibiotic Usage (g/PCU)** | 0.0128 [0.0005**] | 0.0088  [0.0965*] | 0.0066  [0.0145**] | 0.0064  [0.0307**] |

P-values are found in square brackets under model estimates. **Significant at 0.05. * Significant at 0.1. ^NS^ Not Significant.

All fattening pig case studies and the ampicillin-resistance in broiler poultry case study displayed a statistically significant association between antibiotic usage and resistance (p < 0.05). The tetracylcine-resistance in broiler poultry case study was only found to be significant at the p < 0.1 level. Predictions using the regression models and the 95% confidence intervals for model predictions were plotted case study surveillance data (Figure S5).


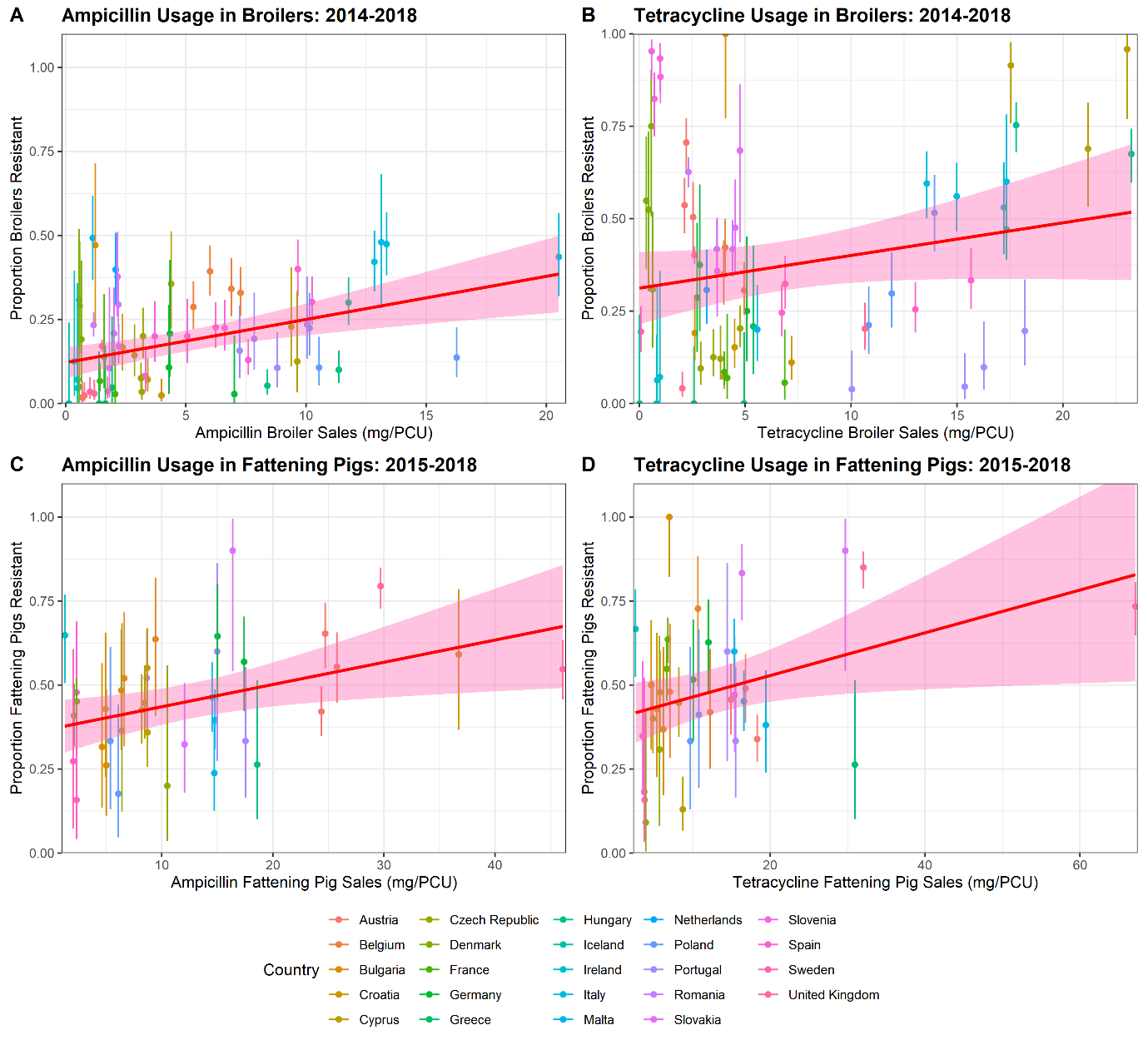


**Figure S5. Relationship between scaled antibiotic sales and the proportion of isolates resistant across different EU country/year pairs from 2014-2018. A) Ampicillin-resistance in broiler poultry, B) tetracycline-resistance in broiler poultry, C) ampicillin-resistance in fattening pigs and D) tetracycline-resistance in fattening pigs.** Solid line and ribbons represent the best fitting linear regression between sales and resistance with 95% CIs for model predictions.

**ABC-SMC Model Fitting**

The ABC-SMC model fit was run for ten generations, with each generation running until the acceptance of 1000 particles. Prior distributions for fitted model parameters, $\theta={[\beta}_{AA},\kappa, \varphi, \alpha,\beta_{HA}, \zeta]$, can be found in Table S3.

**Table S3. Prior distributions used for ABC-SMC model fitting**

| Parameter | Description | Prior Distribution | Description |
| --- | --- | --- | --- |
| **β_AA_** | Per Capita Rate of Transmission (Direct and Indirect) between the Infected Animal Fraction and Susceptible Animal Fraction | $U\left( 0, 0.25 \right)$ | Uninformative  Prior |
| **φ** | Per Capita Rate of Conversion from Antibiotic-Resistant to Antibiotic-Sensitive Infection in Animals | $U\left( 0, 0.1 \right)$ | Uninformative  Prior |
| **κ** | Scaling parameter to model uncertainty in the effects of antibiotic treatment (τ) on the per capita rate of antibiotic-resistant to antibiotic-sensitive conversion. | $U\left( 0, 2 \right)$ | Uninformative  Prior |
| **α** | Transmission-related fitness costs associated with antibiotic-resistance | $Beta(\alpha= 1.5, \beta= 8.5)$ | Vague  Prior |
| **ζ** | Background rate of transmission of foodborne bacteria to the livestock population | $U\left( 0, 1 \right)$  $U\left( 0, 1.5 \right)$ (Ampicillin in broilers) | Uninformative  Prior |
| **β_HA_** | Per Capita Rate of Transmission (Direct and Indirect) between the Infected Animal Fraction and Susceptible Human Fraction | $U\left( 0, 0.0005 \right)$ | Uninformative  Prior |

Acceptance thresholds (ε) were required for each of the three summary statistics for each generation, calculating the difference between the modelled summary statistic and the data. These thresholds can be found in the supplementary material (Table S4).

**Table S4. ε thresholds used for each of the ABC-SMC generations.**

| Summary Statistics | Case Study | Generation | | | | | | | | | |
| --- | --- | --- | --- | --- | --- | --- | --- | --- | --- | --- | --- |
|  |  | **1** | **2** | **3** | **4** | **5** | **6** | **7** | **8** | **9** | **10** |
| **Sum of squared errors** | Ampicillin resistance in Broiler Poultry | 5 | 4 | 3.5 | 3.25 | 3 | 2.75 | 2.5 | 2.25 | 2.1 | 2 |
|  | Tetracycline resistance in Broiler Poultry | 10 | 8 | 7 | 6.5 | 6.25 | 6 | 5.9 | 5.8 | 5.7 | 5.6 |
|  | Ampicillin resistance in Fattening Pigs | 3.5 | 3 | 2.5 | 2 | 1.7 | 1.5 | 1.3 | 1.2 | 1.1 | 1 |
|  | Tetracycline resistance in Fattening Pigs | 3.5 | 3 | 2.5 | 2.25 | 2 | 1.8 | 1.7 | 1.6 | 1.55 | 1.5 |
| **Difference between modelled and observed overall prevalence of human salmonellosis** | Ampicillin resistance in Broiler Poultry | 0.593 | 0.474 | 0.356 | 0.237 | 0.178 | 0.119 | 0.089 | 0.059 | 0.044 | 0.300 |
|  | Tetracycline resistance in Broiler Poultry | 0.593 | 0.474 | 0.356 | 0.237 | 0.119 | 0.059 | 0.047 | 0.412 | 0.036 | 0.300 |
|  | Ampicillin resistance in Fattening Pigs | 0.593 | 0.474 | 0.356 | 0.237 | 0.119 | 0.059 | 0.047 | 0.045 | 0.036 | 0.300 |
|  | Tetracycline resistance in Fattening Pigs | 0.593 | 0.474 | 0.356 | 0.237 | 0.119 | 0.059 | 0.047 | 0.045 | 0.036 | 0.300 |
| **Difference between modelled and observed proportion of resistant human salmonellosis** | Ampicillin resistance in Broiler Poultry | 0.314 | 0.251 | 0.187 | 0.126 | 0.094 | 0.063 | 0.047 | 0.031 | 0.023 | 0.016 |
|  | Tetracycline resistance in Broiler Poultry | 0.316 | 0.253 | 0.189 | 0.126 | 0.063 | 0.032 | 0.025 | 0.022 | 0.019 | 0.015 |
|  | Ampicillin resistance in Fattening Pigs | 0.345 | 0.276 | 0.207 | 0.138 | 0.069 | 0.035 | 0.028 | 0.024 | 0.021 | 0.017 |
|  | Tetracycline resistance in Fattening Pigs | 0.340 | 0.372 | 0.203 | 0.136 | 0.136 | 0.067 | 0.034 | 0.027 | 0.024 | 0.017 |

A multivariate normal distribution was chosen for the ABC-SMC perturbation kernel [16], with the randomly sampled mean and covariance matrix calculated from the previously accepted generation of accepted particles. An intersection metric was used to ensure that accepted particles satisfied tolerance values set for the distance measure for each calculated for each summary statistic per generation. Posterior distributions for the fitted model parameters from the ABC-SMC procedure can be found in Figure S6.


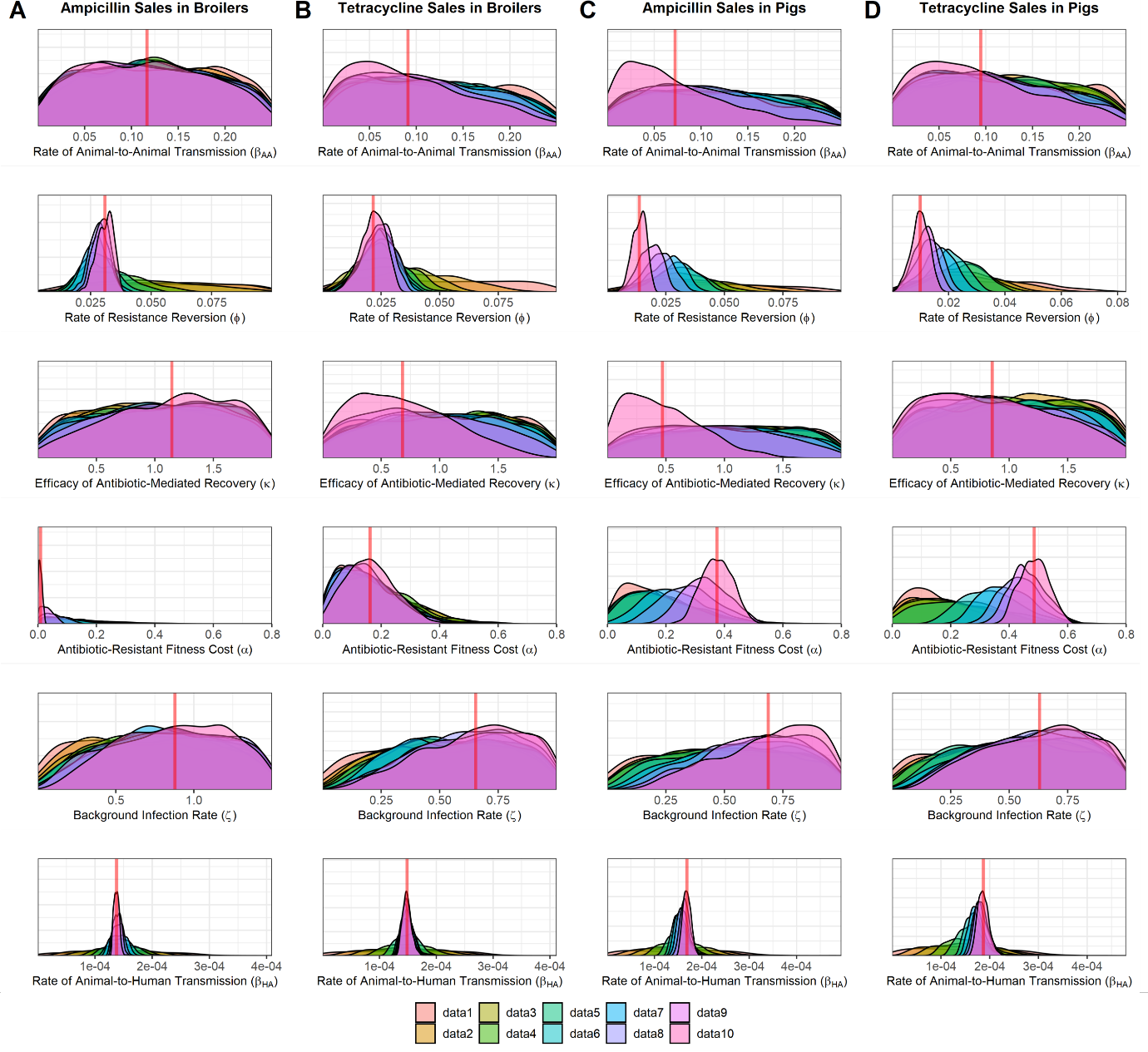


**Figure S6. Estimated posterior distributions for the rate of animal-to-animal transmission (β_AA_), efficacy of antibiotic-mediated recovery (κ), rate of antibiotic-resistant to antibiotic-sensitive reversion (φ), transmission-related fitness costs of resistance (α), background rate of transmission to animal populations (ζ) and the rate of animal-to-human transmission (β_HA_).** A) Ampicillin in broiler poultry, B) tetracycline usage in broiler poultry, C) ampicillin usage in fattening pigs and D) tetracycline usage in fattening pigs. The estimated posterior distribution for each generation is highlighted by fill colours. Red line represents the mean from the 10^th^ generation for each parameter.

Mean point estimates from the approximated marginal posterior probability distributions of the 10^th^ accepted generation were used as the final parameter sets for each respective case study. Point estimates and calculated 95% HDIs from the marginal posterior distribution for each model parameter can be found in the supplementary material (Table S5).

**Table S5. Parameter values for case studies.**

| Parameter | Description | Case Study (Fitted) Parameter Values  (Sum of squares from model fit in square brackets) | | | | References |
| --- | --- | --- | --- | --- | --- | --- |
|  |  | **Ampicillin resistance in Broiler Poultry**  **(SS = 1.987)** | **Tetracycline resistance in Broiler Poultry**  **(SS = 5.473)** | **Ampicillin resistance in Fattening Pigs**  **(SS = 0.969)** | **Tetracycline resistance in Fattening Pigs**  **(SS = 1.456)** |  |
| ***β_AA_*** | Per Capita Rate of Transmission (Direct and Indirect) between the Infected Animal Fraction and Susceptible Animal Fraction | **0.116613 [0.004307,**  **0.227580] ^1^** | **0.091145**  **[0.001947,**  **0.206444] ^1^** | **0.072271 [0.000508,**  **0.178430]^1^** | **0.094618 [0.000415,**  **0.216028] ^1^** | N/A |
| ***β_HH_*** | Per Capita Rate of Transmission (Direct and Indirect) between the Infected Human Fraction and Susceptible Human Fraction | 0.00001 | 0.00001 | 0.00001 | 0.00001 | N/A |
| ***β_AH_*** | Per Capita Rate of Transmission (Direct and Indirect) from the Infected Human Fraction to the Susceptible Animal Fraction | 0.00001 | 0.00001 | 0.00001 | 0.00001 | N/A |
| ***β_HA_*** | Per Capita Rate of Transmission (Direct and Indirect) from the Infected Animal Fraction to the Susceptible Human Fraction | **0.0001388**  **[0.000130,**  **0.000145] ^1^** | **0.000149**  **[0.000134,**  **0.000163] ^1^** | **0.000169**  **[0.000154,**  **0.000184] ^1^** | **0.0001870**  **[0.000166,**  **0.000210] ^1^** | N/A |
| ***ζ*** | Background rate of transmission of foodborne bacteria to the livestock population | **0.8789726 [0.285250,**  **1.478116] ^1^** | **0.653686**  **[0.250350,**  **0.999701] ^1^** | **0.687408 [0.302243,**  **0.998396] ^1^** | **0.629688 [0.239937,**  **0.997063] ^1^** | N/A |
| ***τ*** | Per Capita Rate of Antibiotic Usage in Livestock (Baseline) in g/PCU | 0.0067 | 0.0067 | 0.0123 | 0.0116 | N/A |
| *κ* | Efficacy of antibiotic-mediated livestock recovery. | **1.144771 [0.251745,**  **1.995368] ^1^** | **0.682709**  **[0.006160,**  **1.459921] ^1^** | **0.470487 [0.000743,**  **1.058426] ^1^** | **0.854826 [0.018652,**  **1.793183] ^1^** | N/A |
| ***α*** | Transmission-related fitness costs associated with antibiotic-resistant strains (relative to antibiotic-sensitive strains). | **0.009010 [0.000461,**  **0.020193] ^1^** | **0.162060**  **[0.011241,**  **0.317247] ^1^** | **0.374363 [0.281892,**  **0.474565] ^1^** | **0.485538 [0.391098,**  **0.572014] ^1^** | N/A |
| ***φ*** | Per Capita Rate of Conversion from antibiotic-resistant to antibiotic-sensitive infection in animals | **0.030938**  **[0.024368,**  **0.036523] ^1^** | **0.021652 [0.011457,**  **0.031297] ^1^** | **0.013677 [0.008160,**  **0.019323] ^1^** | **0.009931 [0.004160,**  **0.016450] ^1^** | N/A |
| ***r_A_*** | Per Capita Rate of Natural Recovery from Animal Infection | 0 days^-1^ | 0 days^-1^ | 60 days^-1^ | 60 days^-1^ | [17] |
| ***r_H_*** | Per Capita Rate of Natural Recovery from Human Infection | 5.5 days^-1^ | 5.5 days^-1^ | 5.5 days^-1^ | 5.5 days^-1^ | [18] |
| ***µ_A_*** | Per Capita Birth/Death Rate in Animals | 42 days^-1^ | 42 days^-1^ | 240 days^-1^ | 240 days^-1^ | [19] |
| ***µ_H_*** | Per Capita Birth/Death Rate in Humans | 28835 days^-1^ | 28835 days^-1^ | 28835 days^-1^ | 28835 days^-1^ | [20] |

^1^Note that values in bold are mean point estimates from the posterior distribution of fitted parameters, lower and upper bounds of the 95% HDI are shown in square brackets.

Observed country-level antibiotic usage and livestock tetracycline/ampicillin-resistance surveillance data was plotted for all four case studies, with the model output overlaid (Figure S7). It is important to note that the ζ parameter (ζ > 0) is necessary to prevent I^*^_RHProp_ decreasing to 0 upon livestock antibiotic curtailment (τ = 0 g/PCU). Inclusion of the ζ parameter was shown to provide a better fit to the model compared to a null model with ζ = 0 (Figure S18).


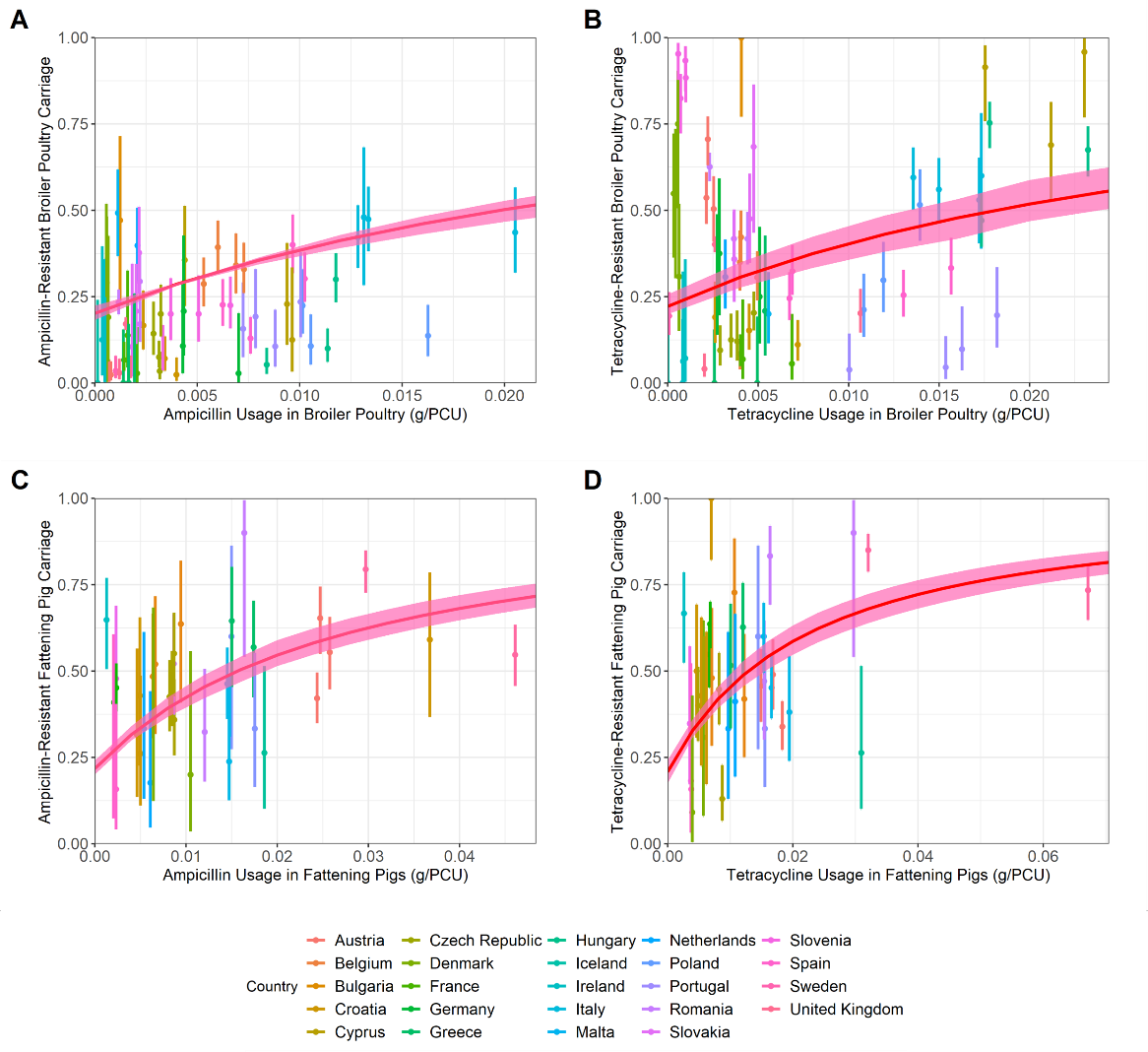


**Figure S7. Observed and estimated relationship between livestock antibiotic usage and the prevalence of antimicrobial-resistant salmonellosis in humans. A) Ampicillin-resistance in broiler poultry, B) tetracycline-resistance in broiler poultry, C) ampicillin-resistance in fattening pigs and D) tetracycline-resistance in fattening pigs.** Solid red lines and ribbons represent model fit resulting from the approximated posterior distribution using ABC-SMC and the corresponding 95% HDI. Country-specific 95% confidence intervals for the observed data (dots) were calculated for each case study using a 1-sample proportion test with continuity correction.

The outcome measures resulting from the fitted model parameters for the four case studies can be found in Table S6. These values were calculated for the baseline values of antibiotic usage for the case studies.

**Table S6. Fitted values for the primary outcome measures for the four model case studies.**

| Outcome Measure | Case Study | | | |
| --- | --- | --- | --- | --- |
|  | **Ampicillin Resistance in Broiler Poultry**  **[0.0049 g/PCU]** | **Tetracycline Resistance in Broiler Poultry**  **[0.0069 g/PCU]** | **Ampicillin Resistance in Fattening Pigs**  **[0.0125 g/PCU]** | **Tetracycline Resistance in Fattening Pigs**  **[0.01305 g/PCU]** |
| Daily incidence of human salmonellosis | 0.595 | 0.600 | 0.598 | 0.601 |
| Proportion of antibiotic-resistant human salmonellosis  (I^*^_RHProp_) | 0.301 | 0.356 | 0.349 | 0.342 |

Baseline antibiotic usage displayed in square brackets for each case study.

**Model Comparison and ζ Parameter**

We note that the addition of the ζ parameter was done to prevent the fraction of antibiotic-resistant human infection (I^*^­_RHProp_) descreasing to 0 upon total curtailment of livestock antibiotic (τ = 0.00934 → 0 g/PCU). Using the ABC-SMC framework, we can undertake a formal comparison to identify if the addition of this ζ parameter performs better than the nested null hypothesis model where ζ = 0, and I^*^­_RHProp_ is initiated at the origin when livestock antibiotic usage is curtailed (Toni et al, 2009) [16].

We define a new parameter $m$ describing the model choice, with $m\in\{m_{1},m_{2}\}$ and $m_{1}$ and $m_{2}$ corresponding to the ODEs described in eqn 1.1, where $\zeta>0$ in $m_{1}$ and $\zeta=0$ in $m_{2}$, and with $m_{2}$ is nested within $m_{1}$. We note that the overall aim of the model selection approach identified in Toni et al, 2009, is to provide an approximation of the marginal posterior distribution of the parameter $m$given the data, $P(m|x)$ [16]. Model specific parameter vectors are then created, $\theta(m)$, with only the fitted parameters represented: $\theta\left( 1 \right)=[\beta_{AA},\varphi,\alpha,\kappa, \beta_{HA},\zeta,m_{1}]$ and $\theta\left( 2 \right)=[\beta_{AA},\varphi,\alpha,\kappa, \beta_{HA},m_{2}]$. The prior distributions used in the model comparison approach are identical to those used to fit the model parameters (Table S3), with a discrete uniform distribution limited at 1 and 2, used for the model selection parameter, $m=U(1,2)$. The model comparison algorithm is detailed in Toni et al, 2009 [16].

This Bayes factor is a summary of the evidence for one model over the other given the data. We can recover the equation for the Bayes factor through an odds transformation of the marginal posterior probability of $m_{1}$ given the data and $m_{2}$ given the data (eqn S2.1) [21].

$$\frac{P(m_{1}|x)}{P(m_{2}|x)}=\frac{P(x|m_{1})}{P(x|m_{2})}\frac{P(m_{1})}{P(m_{2})}$$

Eqn S2.1

With the Bayes factor, $B_{12}$, being (eqn S2.2):

$$B_{12}=\frac{P(x|m_{1})}{P(x|m_{2})}$$

Eqn S2.2

If we assume that the prior distribtions for $m_{1}$ and $m_{2}$ are uniform, then we can cancel the last multiplicative term in eqn S2.1, and therefore we recover the equation for $B_{12}$ by substituting eqn S2.2 in eqn S2.1.

$$B_{12}=\frac{P(m_{1}|x)}{P(m_{2}|x)}$$

Eqn S2.3

The Bayes factor is therefore a ratio of the posterior probability of $m_{1}$ given the data and $m_{2}$ given the data. As the ABC-SMC algorithm returns an approximation of the marginal posterior distribution of the $m_{1}$ and $m_{2}$, $P(m_{1}|x)$ and $P(m_{2}|x)$, we can simply take a ratio of the number of accepted particles for each model in the last generation. This represents the model with the highest posterior probability. This is therefore an approximation of the Bayes factor and allows for model selection. Therefore, we denote the model with the greatest number of accepted particles in the last generation, the best fitting model. We run the model fitting process until 10 generations of 1000 accepted particles, or until one model is the sole model structure chosen. As stated in Toni et al, 2009, the model selection algorithm implicitely penalizes models with a large number of parameters, as models with a larger parameter dimension have a smaller probability of being accepted [16].


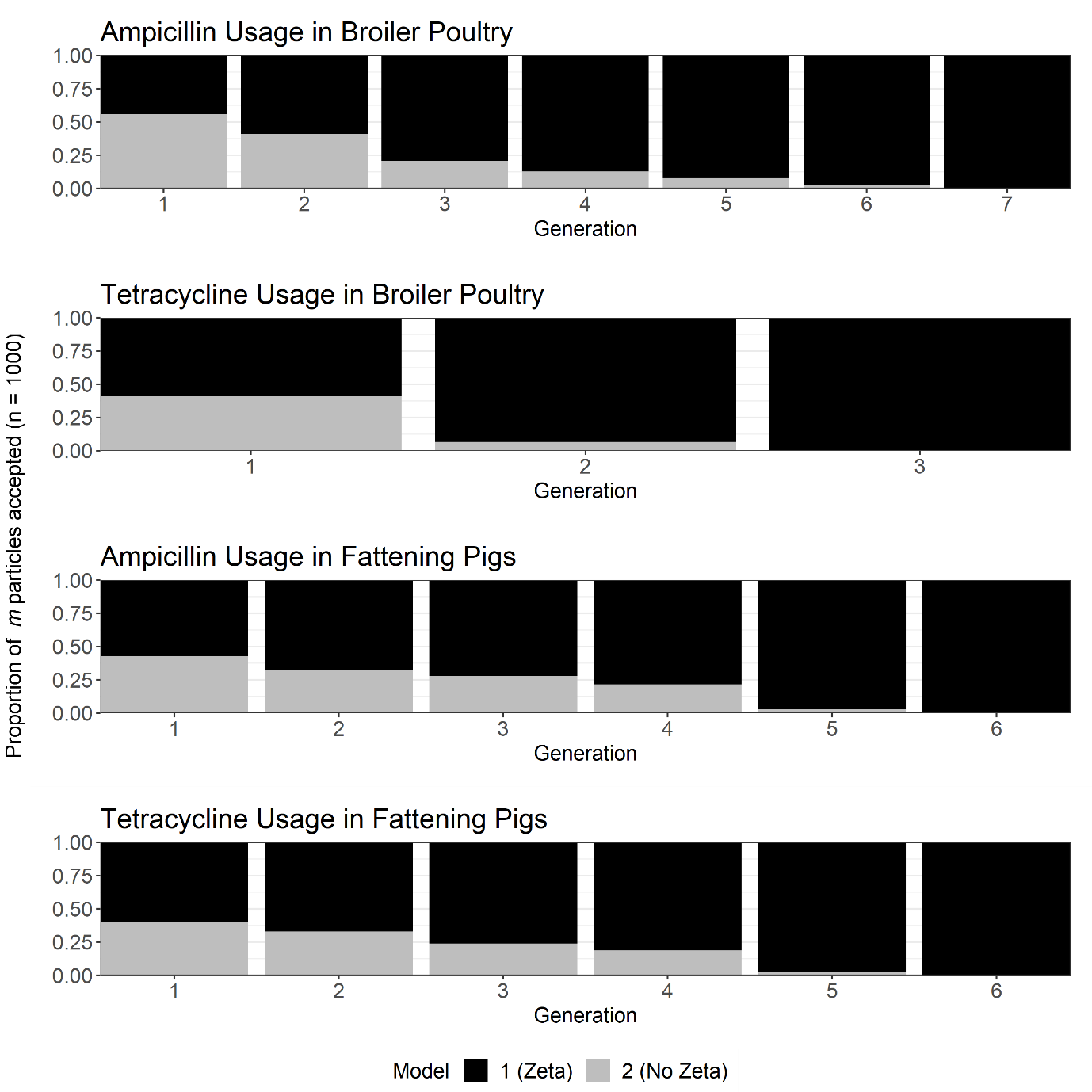


**Figure S8**. Ratio of the accepted particles for $P(m_{1}|x)$ and $P(m_{2}|x)$, across all generations of the ABC-SMC model selection for the ampicillin usage in broiler poultry, tetracycine usage in broiler poultry, ampicillin usage in fattening pigs and tetracycline usage in fattening pigs case studies.

We find that all case studies result in a model “die-out” with model 1 ($m_{1}$) being the sole selected model in the last generation (Figure S8). Note that in the initial generations for the ampicillin resistance in broiler poultry case study, model selection favored $m_{2}$, settling on $m_{1}$ as $m$ reaches the final posterior distribution at $G=7$. As described in Toni et al, 2009, this is likely due to the selection algorithm passing a local maximum favoring $m_{2}$ on the way to $m_{1}$ [16].

**ABC-SMC diagnostics from model fitting**

We next assessed the diagnostics of the model fit by looking at the average sum of squared distance, average relative distance from the daily incidence and target human resistance for each generation across each case study (Figure S9). We note that intuitively, there is a downwards trajectory for all summary statistics bar the ampicillin usage in broiler poultry case study, demonstrating that for the other three case studies each generation is fitting closer to the desired value. We note that this plateau in the sum of squared distances past the 5^th^ generation is likely due to the tightening in ε tolerances for the distance from the target proportion of human resistance. This can be observed with a minima observed generation 2-3 for the sum of squared distances, which corresponds with a maxima for the distance from the target proportion of human resistance. This suggests that there is tension between these two summary statistics.


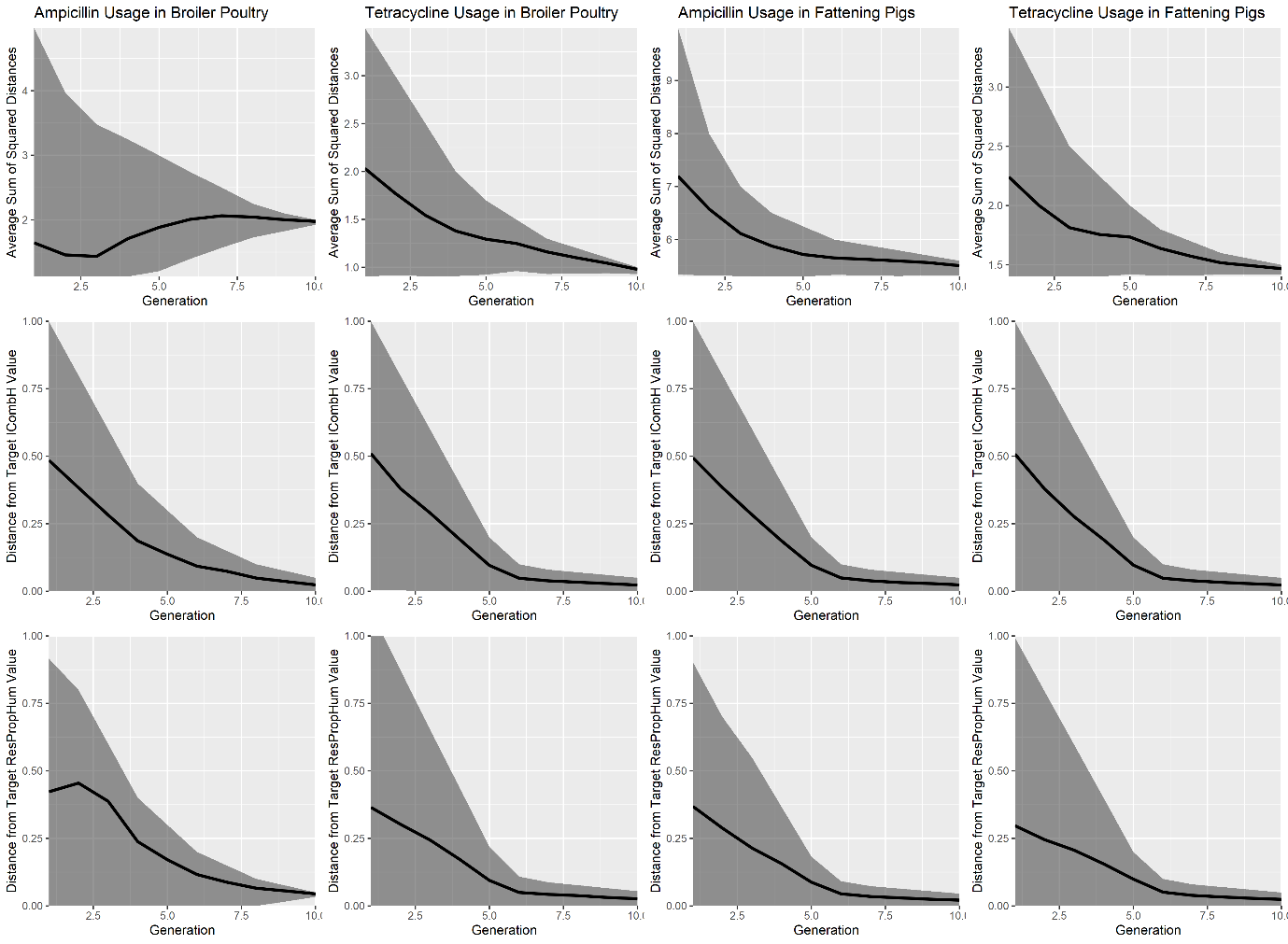


**Figure S9**. Diagnostic plots showing the average sum of squared distance for each generation (g set of 1,10)

Pairs plots were also generated for each case study, with the approximated joint posterior distribution and correlation coefficients populated for each parameter combination (Figure S10-13).


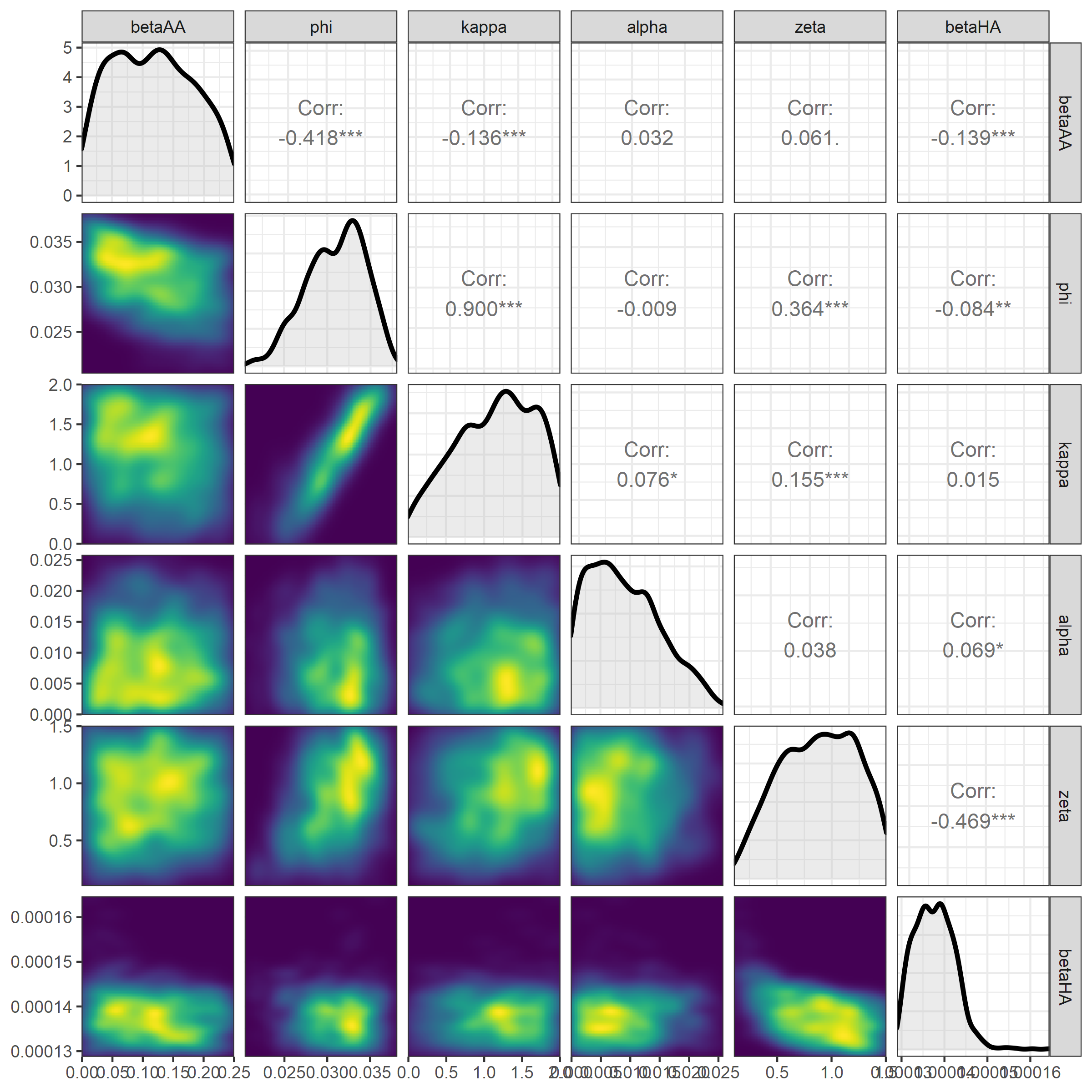


**Figure S10. Pairs plot for the ampicillin resistance in broiler poultry case study showing the approximated joint posterior distribution and correlation coefficients with the diagonals showing the approximated univariate posterior distribution.** Kernel density estimation was used to identify the parameter space where a greater concentration of particles were accepted for the final tenth ABC-SMC generation (lighter colouring).


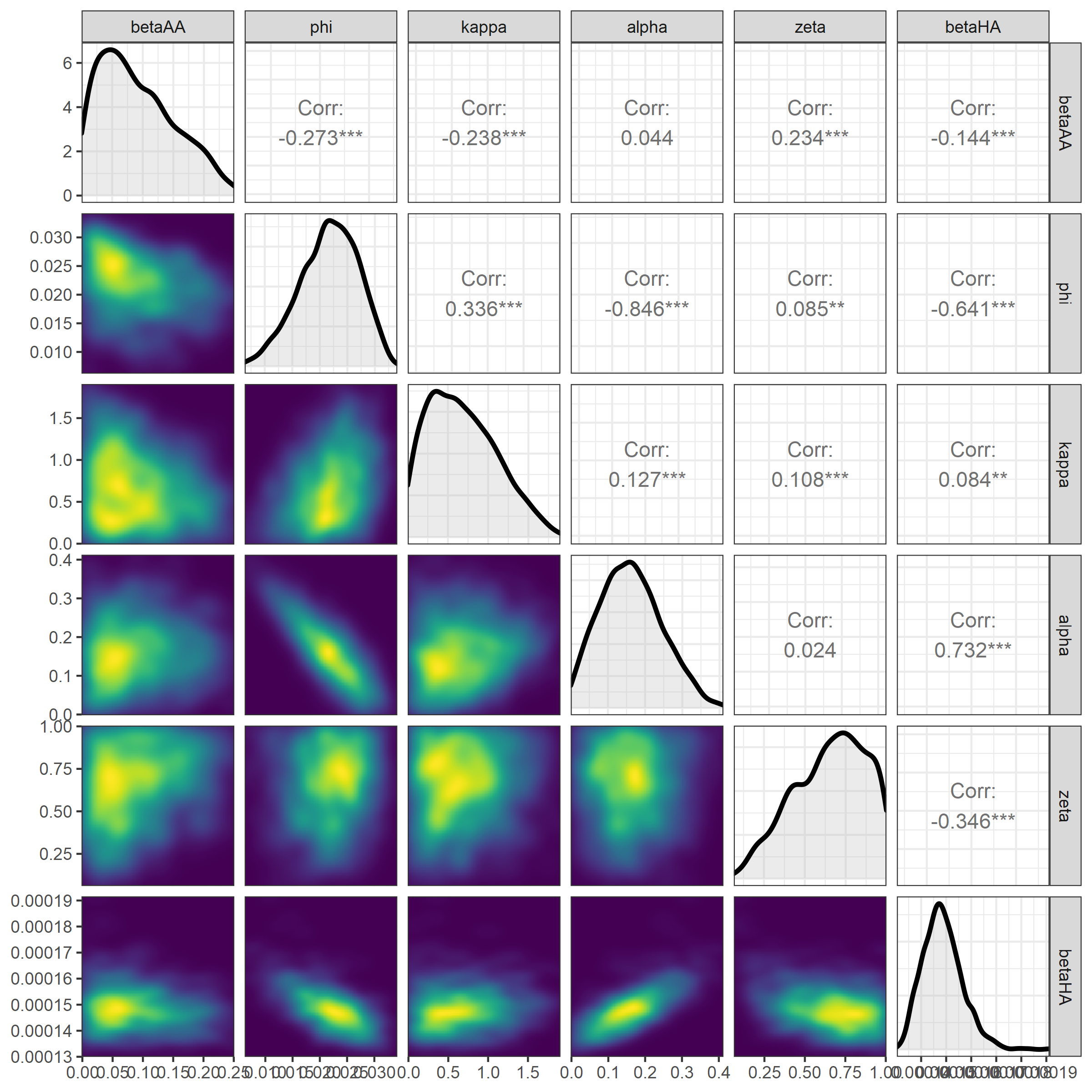


**Figure S11**. **Pairs plot for the tetracycline resistance in broiler poultry case study showing the approximated joint posterior distribution and correlation coefficients with the diagonals showing the approximated univariate posterior distribution.** Kernel density estimation was used to identify the parameter space where a greater concentration of particles were accepted for the final tenth ABC-SMC generation (lighter colouring).


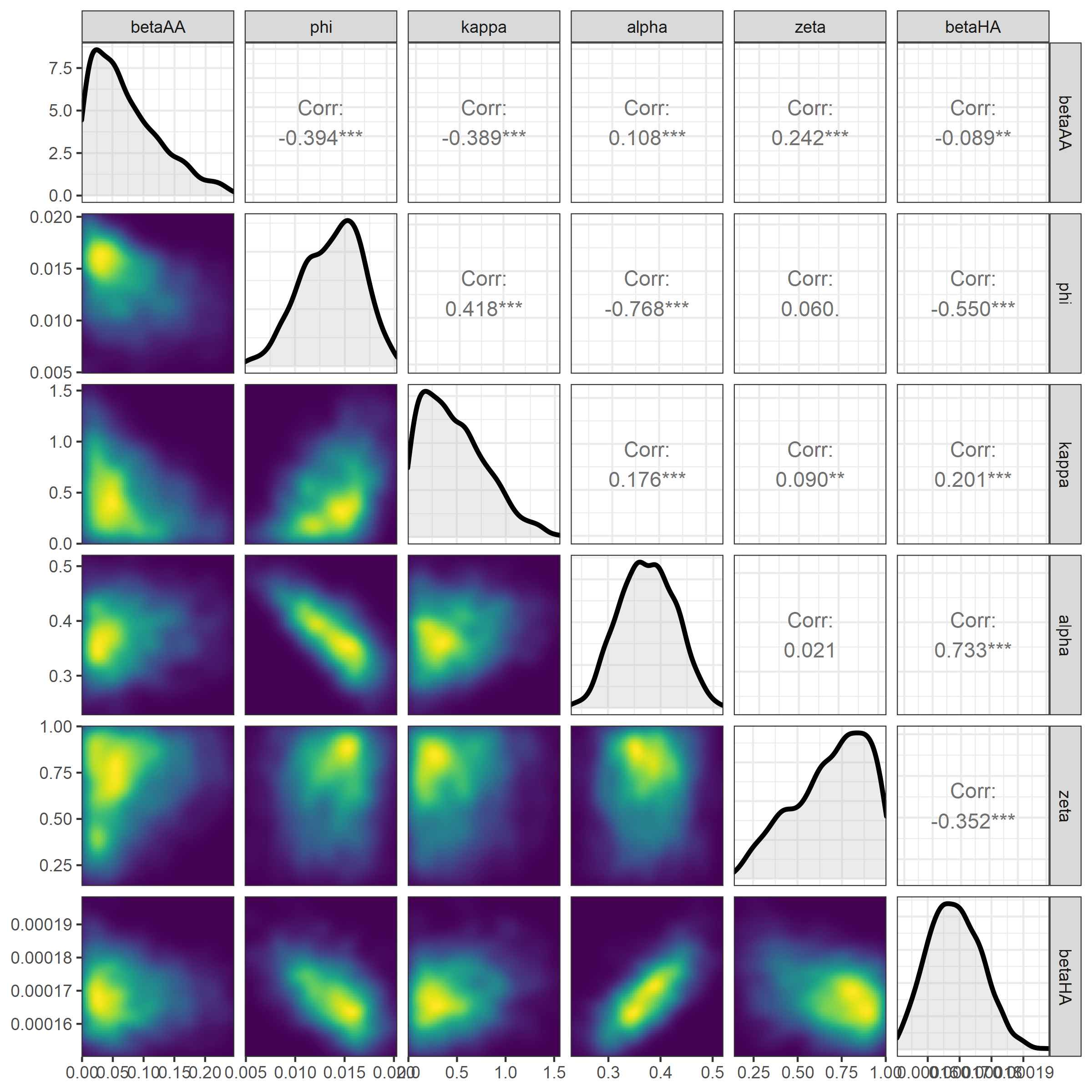


**Figure S12.** **Pairs plot for the ampicillin resistance in fattening pigs case study showing the approximated joint posterior distribution and correlation coefficients with the diagonals showing the approximated univariate posterior distribution.** Kernel density estimation was used to identify the parameter space where a greater concentration of particles were accepted for the final tenth ABC-SMC generation (lighter colouring).


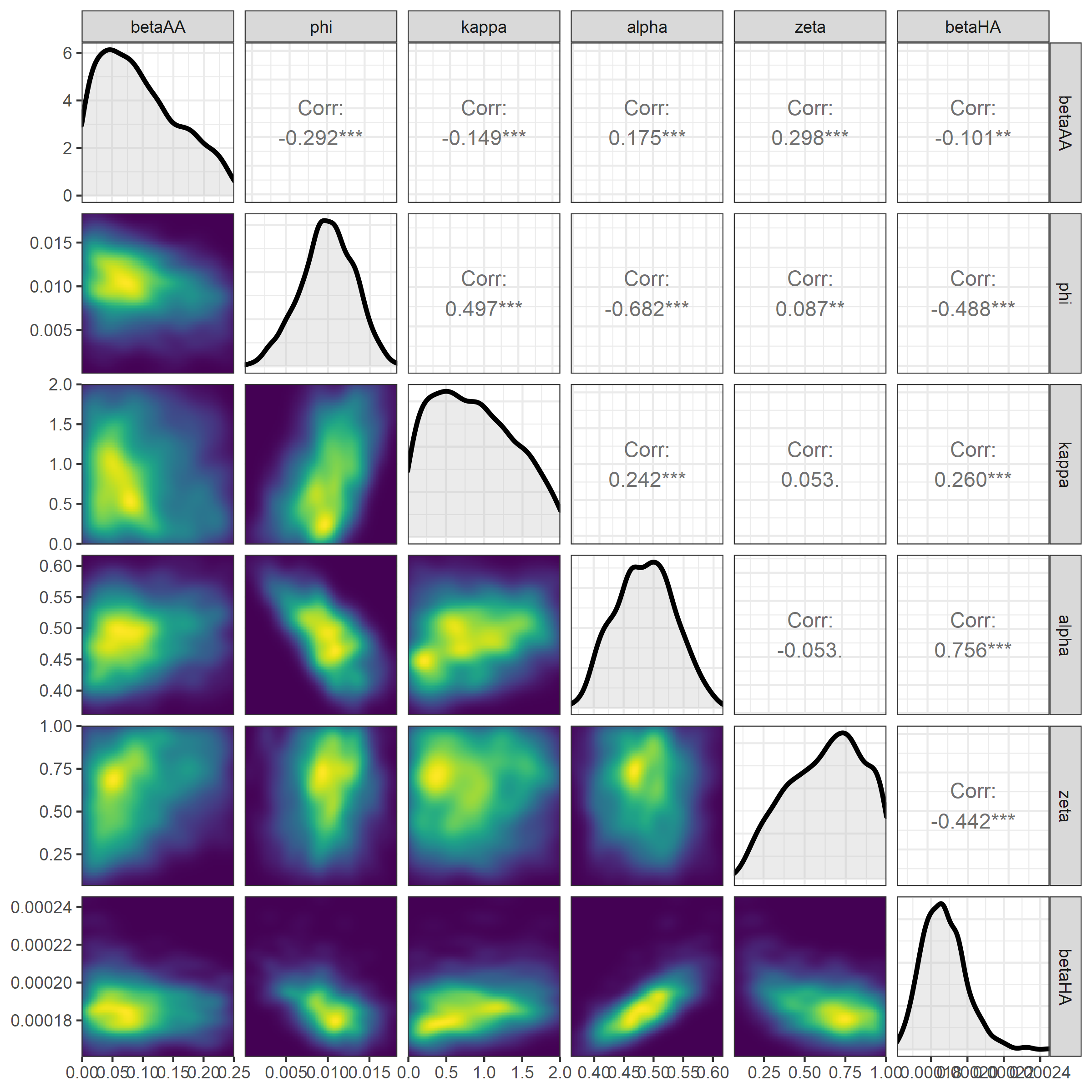


**Figure S13. Pairs plot for the tetracycline resistance in fattening pigs case study showing the approximated joint posterior distribution and correlation coefficients with the diagonals showing the approximated univariate posterior distribution.** Kernel density estimation was used to identify the parameter space where a greater concentration of particles were accepted for the final tenth ABC-SMC generation (lighter colouring).

**Fourier Amplitude Sensitivity Test analyses**

The Fourier amplitude sensitivity test (FAST) is a variance-based sensitivity analysis that partitions variance in the model output to variation in the model parameters [22]. It does so through the calculation of Fourier coefficients at different frequencies corresponding to the identify of unique model parameters. We explored the sensitivity of two main outcome measures to the model parameters (with regards to livestock antibiotic curtailment):

1. *Relative changes in the daily incidence when livestock antibiotics are curtailed (τ =* 0 *g/PCU), compared to the daily incidence at the baseline livestock antibiotic usage (τ =* 0.00934  *g/PCU).*

The purpose of this outcome measure was to identify parameters (excluding τ) which have the greatest influence on relative changes in the daily incidence when livestock antibiotics are curtailed from baseline levels (τ = 0.00934 → 0 g/PCU). We note that this outcome measure allows for the baseline level of the daily incidence to change with each combination of parameters from the Fourier sampling algorithm, with each scenario possessing a unique baseline level of the daily incidence at τ = 0.00934 g/pCU, with alterations to other model parameters reflecting a new location/drug/bug livestock host scenario or case study. By assuming this flexible baseline level of the daily incidence, we can explore parameters or scenarios which will result in the greatest relative change in daily incidence when livestock antibiotics are curtailed (*τ* = 0 g/PCU). The baseline level of livestock antibiotic usage was fixed at τ = 0.00934 g/PCU to facilitate the comparison of outcome measures across the different parameter combinations. The outcome measure is formally defined as: daily incidence at τ = 0 / daily incidence at τ = 0.00934.

1. *Relative changes in daily incidence when livestock antibiotics were curtailed (τ = 0 g/ PCU), compared to daily incidence of 0.593 per 100,000 population.*

This outcome measure allows for the identification of parameters (excluding τ) which can best control increases in daily incidence upon livestock antibiotic curtailment (*τ* = 0 g/PCU). This is similar to the previous outcome measure, but with the daily incidence at baseline livestock usage fixed to 0.593 per 100,000 population, representing the baseline level of daily incidence for the four considered case studies. This fixed value can be considered a threshold of daily incidence that would be undesirable to exceed, due to this being the current levels of daily incidence observed at baseline livestock antibiotic usage levels (*τ* = 0.00934 g/PCU). By fixing the daily incidence and identifying relative variation from this “threshold” value, we can identify parameters that result in the greatest change from this threshold, and by extension parameters which can best control or prevent increases in the daily incidence beyond what we already observe with livestock antibiotic usage. The outcome measure is formally defined as: daily incidence at τ = 0 g/PCU / 0.593 per 100,000.

**Software Used**

All simulations were carried out using R and RStudio. R package “rootSolve” was used for all model simulations [23]. All sensitivity analyses were performed using the fast and sensitivity R packages [24]. The ABC-SMC approach used “tmvtnorm” and “bayestestR” packages [25, 26]. Plotting used “ggplot2”, “ggpubr”, “metR” and “gridExtra” R packages [27-30].

**Supplementary Figures**


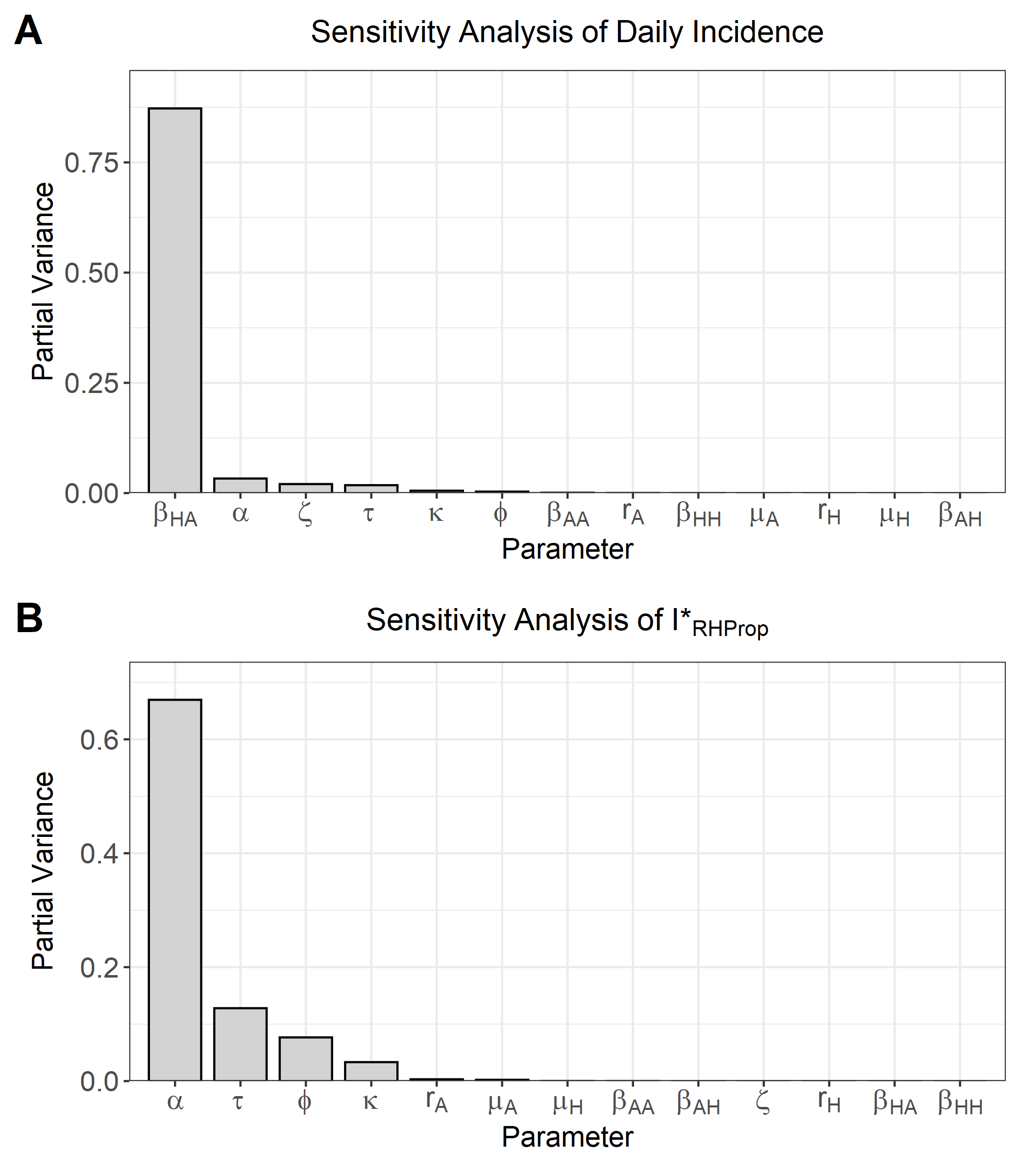


**Figure S14. Fourier amplitude senstivity test (FAST) to identify the most influential model parameter for: A) EU-averaged daily incidence of non-typhoidal salmonellosis. B) The fraction of antibiotic-resistant human non-typhoidal salmonellosis (I^*^­_RHProp_).** Parameters for each sensitivity analysis are ordered from left-to-right by the most influential model parameter (partial variance) for the respective analysis.


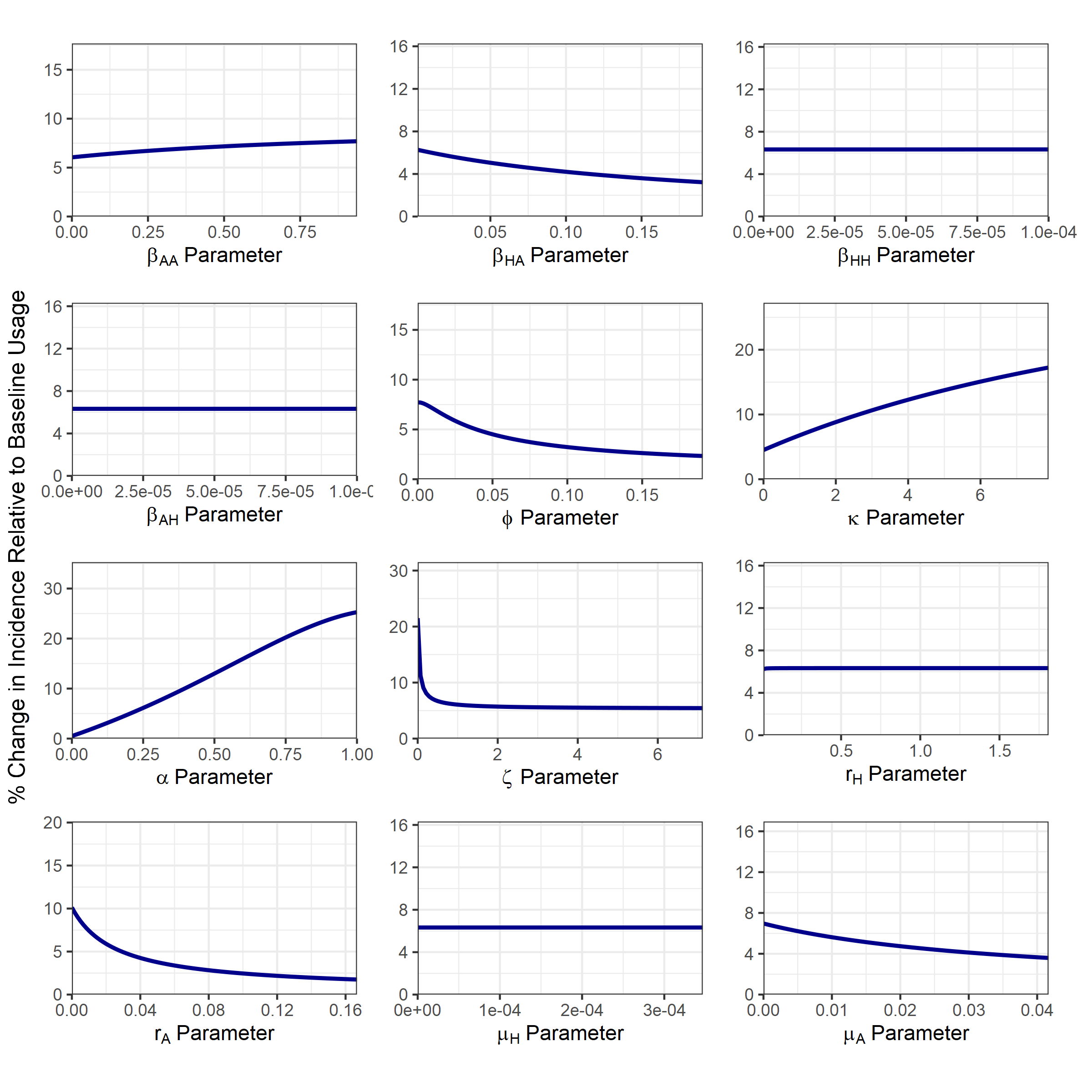


**Figure S15. Impact of varying each model parameter individually on the percentage change in daily incidence under livestock antibiotic curtailment (τ = 0 g/PCU) relative to mean baseline livestock antibiotic usage across the four case studies (τ = 0.00934 g/PCU).** It is important to note that a % change can be interpreted as a relative increase or decrease relative to baseline daily incidence. The direction of the relative change is described in the main text. The explored parameter range for each parameter was bounded at 0 to an order of magnitude above the parameterised model value. An exception was for *r_H_*, with *r_H_* ∈ [0.01, 0.55^-1^] to prevent the large relative changes in daily incidence at *r_H_* = 0 obscuring presented results. For fitted parameter this was taken as an order of magnitude above the mean fitted parameter value across all four case studies.


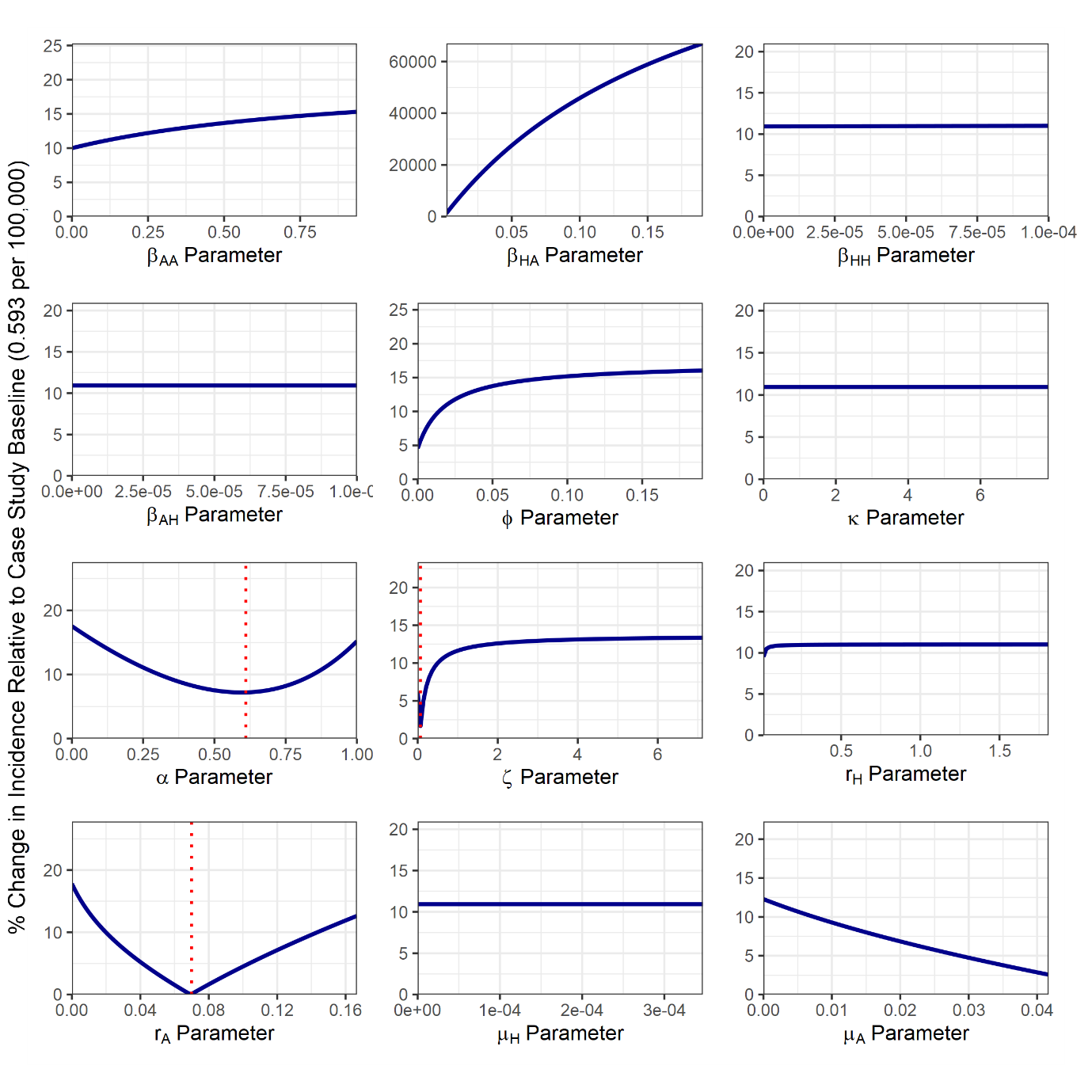


**Figure S16. Impact of varying each model parameter individually on the percentage change in daily incidence under livestock antibiotic curtailment (τ = 0 g/PCU) relative to the baseline daily incidence under current levels of antibiotic usage 0.593 per 100,000 population.** Note that the red, dotted line represents parameters which have both a relative increase and decrease in daily incidence from the baseline threshold of 0.593 per 100,000 population representing a non-monotonic relationship with the outcome measure. Note that the only non-monotonic relationships were found with α, ζ and r_A_. The explored parameter range for each parameter was bounded at 0 to an order of magnitude above the parameterised model value. An exception was for *r_H_*, with *r_H_* ∈ [0.01, 0.55^-1^] to prevent the large relative changes in the daily incidence at *r_H_* = 0 obscuring presented results. For fitted parameter this was taken as an order of magnitude above the mean fitted parameter value across all four case studies.


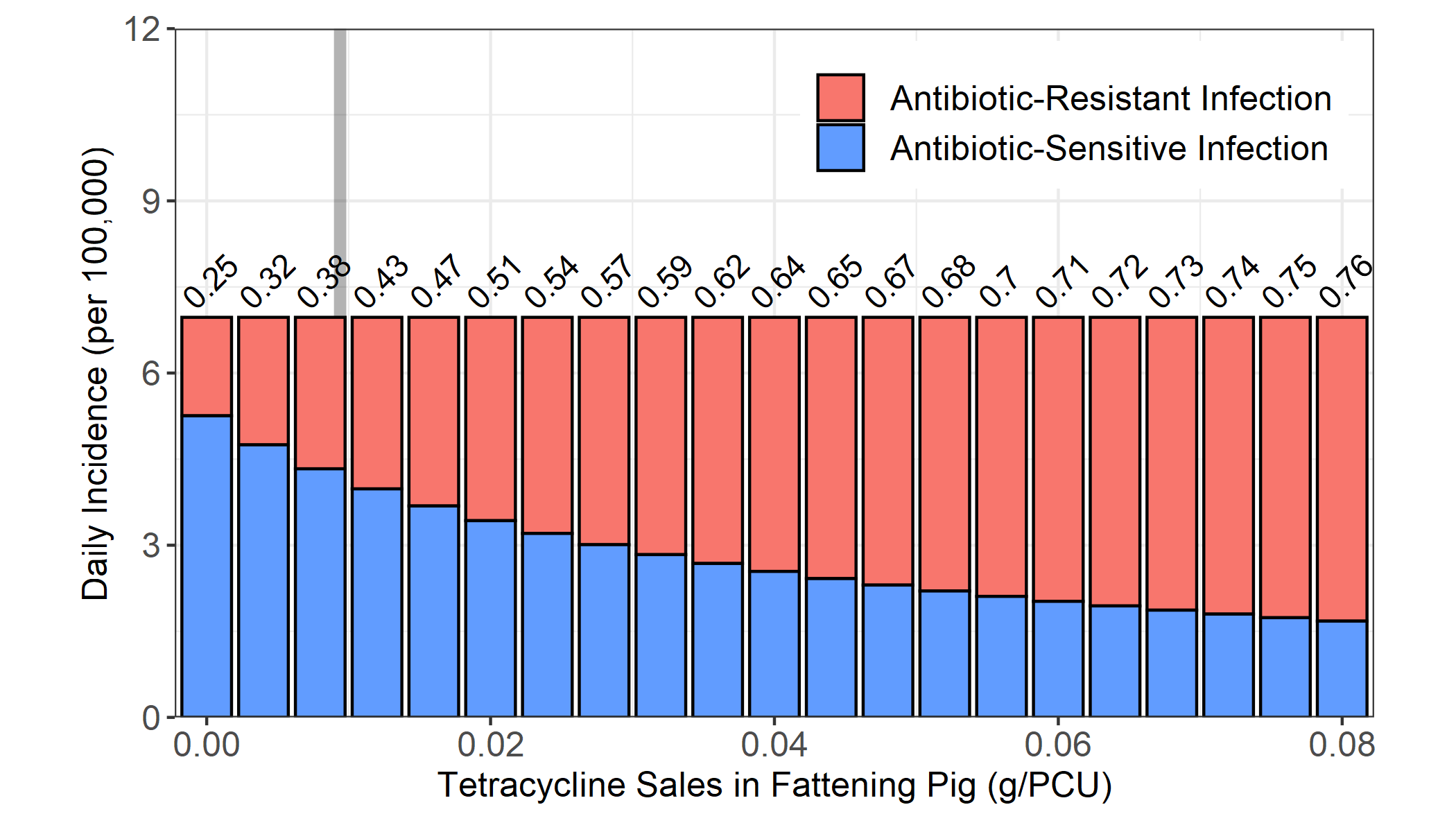


**Figure S17. Impact of alterations in livestock antibiotic sales (τ) on the daily incidence of salmonellosis and the proportion of resistant human infection (I^*^_RHProp_).** Grey line represents the averaged baseline antibiotic usage across all four case studies (0.00934 g/PCU). Parameters α and κ were set to 0 as an illustrative example. This represents a scenario where livestock antibiotics have no therapeutic effect in livestock and fitness costs of resistance have no effect on transmission. Reductions to livestock antibiotic usage now has no discernible effect on increasing human foodborne disease (τ).


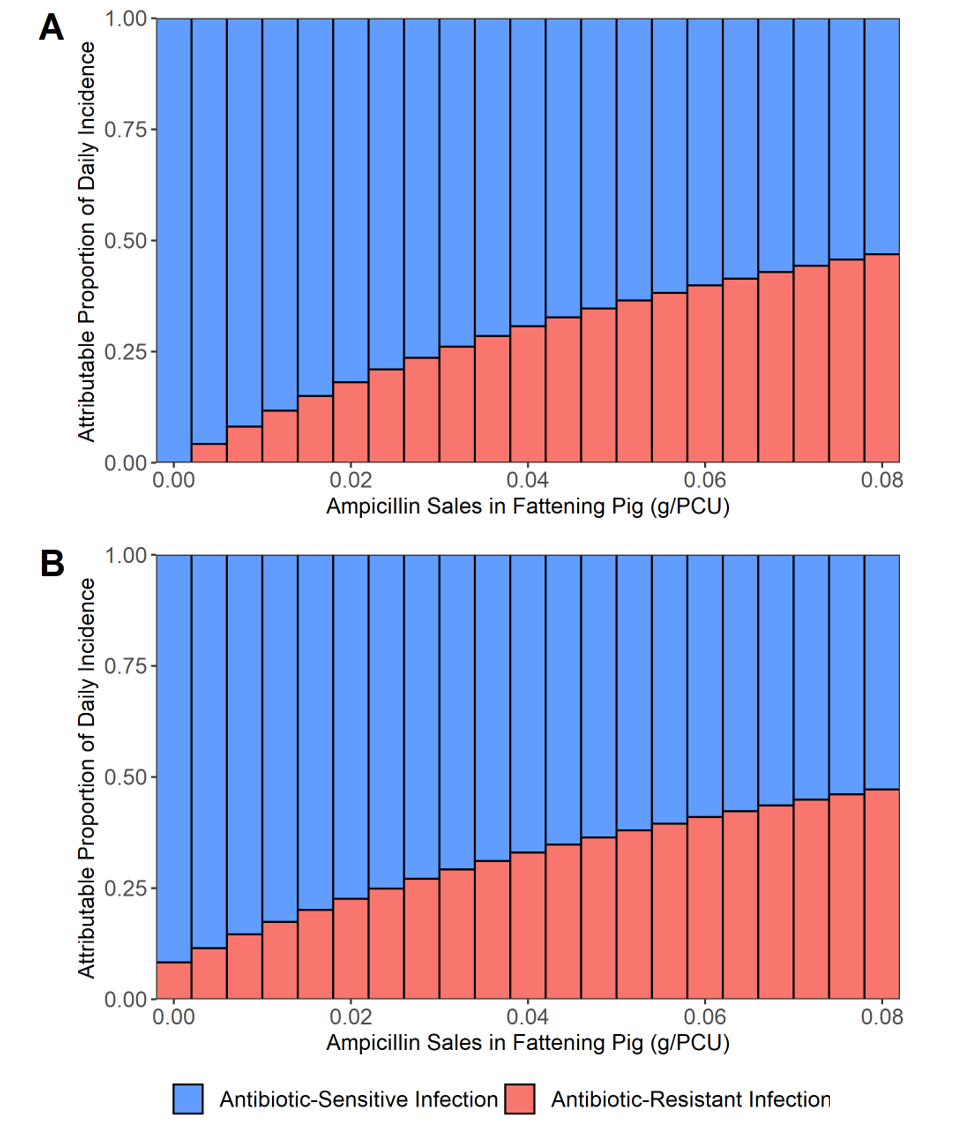


**Figure S18. Impact of alterations on the ζ parameter on the relationship between livestock antibiotic sales (τ) and the proportion of daily incidence attributable to antibiotic-sensitive and antibiotic resistant infection. A) No background transmission to livestock populations (ζ = 0). B) Background transmission to livestock populations (ζ =** **1.328).** The value for the ζ parameter was taken from the mean value of ζ across the four fitted model case studies.


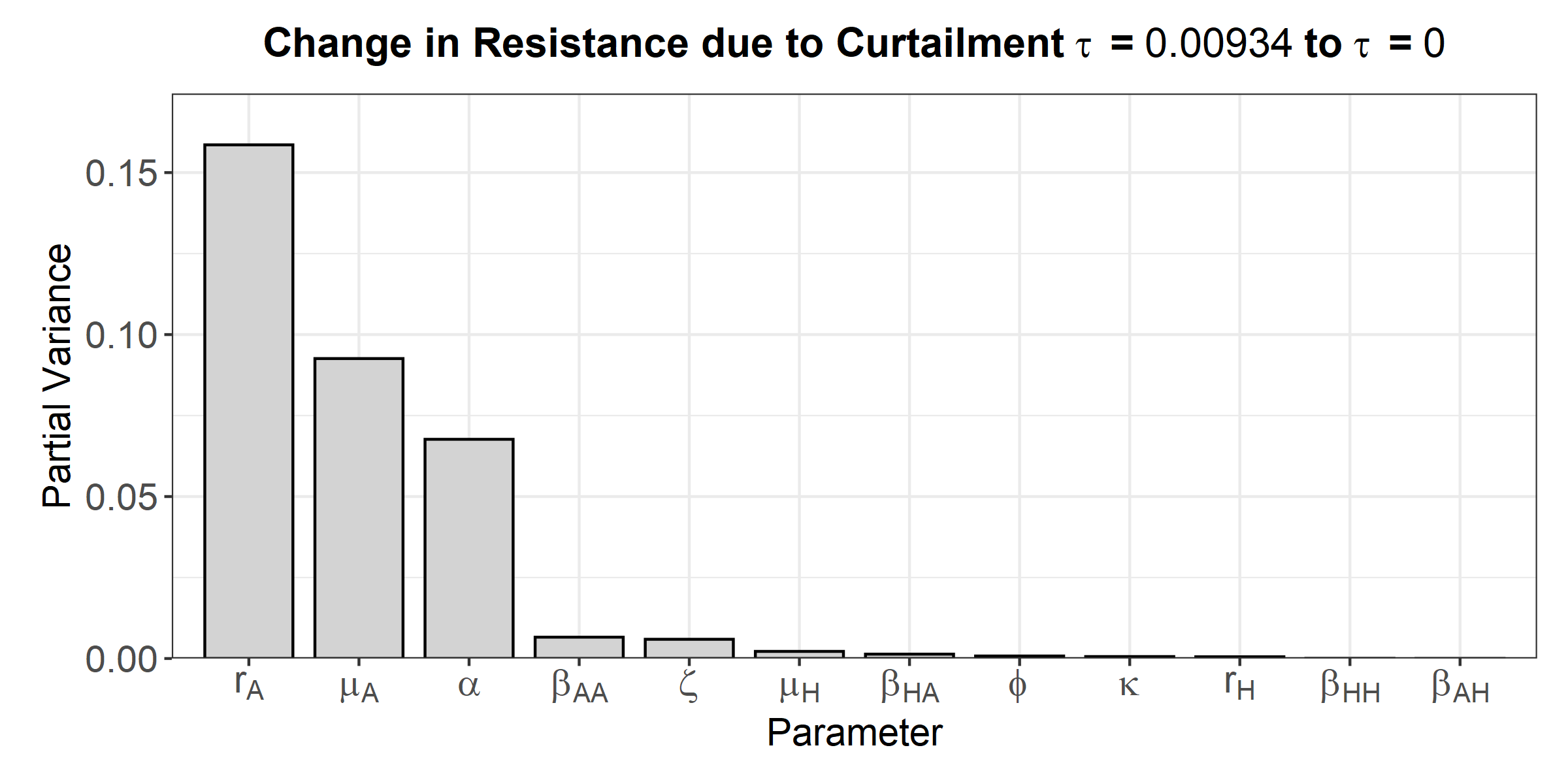


**Figure S19. Fourier amplitude sensitivity test (FAST) to identify the most influential model parameter for the relative change in the proportion of resistant human infection (I^*^_RHProp_) under curtailment (0 g/PCU) compared to the averaged baseline antibiotic usage level (0.00934 g/PCU).**

**Supplementary References**

1. European Medicines Agency, European Surveillance of Veterinary Antimicrobial Consumption. *Sales of veterinary antimicrobial agents in 31 European countries in 2014*. European Medicines Agency. (2016). Available from: <https://www.ema.europa.eu/en/documents/report/sixth-esvac-report-sales-veterinary-antimicrobial-agents-29-european-countries-2014_en.pdf>.

2. European Medicines Agency, European Surveillance of Veterinary Antimicrobial Consumption. *Sales of veterinary antimicrobial agents in 31 European countries in 2015*. European Medicines Agency. (2017). Available from: <https://www.ema.europa.eu/en/documents/report/seventh-esvac-report-sales-veterinary-antimicrobial-agents-30-european-countries-2015_en.pdf>.

3. European Medicines Agency, European Surveillance of Veterinary Antimicrobial Consumption. *Sales of veterinary antimicrobial agents in 31 European countries in 2016*. European Medicines Agency. (2018). Available from: <https://www.ema.europa.eu/en/documents/report/sales-veterinary-antimicrobial-agents-30-european-countries-2016-trends-2010-2016-eighth-esvac_en.pdf>.

4. European Medicines Agency, European Surveillance of Veterinary Antimicrobial Consumption. *Sales of veterinary antimicrobial agents in 31 European countries in 2017*. European Medicines Agency. (2019). Available from: <https://www.ema.europa.eu/en/documents/report/sales-veterinary-antimicrobial-agents-31-european-countries-2017_en.pdf>.

5. European Medicines Agency, European Surveillance of Veterinary Antimicrobial Consumption. *Sales of veterinary antimicrobial agents in 31 European countries in 2018*. European Medicines Agency. (2020). Available from: <https://www.ema.europa.eu/en/documents/report/sales-veterinary-antimicrobial-agents-31-european-countries-2018-trends-2010-2018-tenth-esvac-report_en.pdf>.

6. European Food Safety Authority, Prevention ECfD, Control. The European Union summary report on antimicrobial resistance in zoonotic and indicator bacteria from humans, animals and food in 2014. *EFSA journal*. (2016). 14(2):4380.

7. European Food Safety Authority, Prevention ECfD, Control. The European Union summary report on antimicrobial resistance in zoonotic and indicator bacteria from humans, animals and food in 2015. *EFSA Journal*. (2017). 15(2):e04694.

8. European Food Safety Authority, Prevention ECfD, Control. The European Union summary report on antimicrobial resistance in zoonotic and indicator bacteria from humans, animals and food in 2016. *EFSA Journal*. (2018). 16(2):e05182.

9. European Food Safety Authority, Prevention ECfD, Control. The European Union summary report on antimicrobial resistance in zoonotic and indicator bacteria from humans, animals and food in 2017. *EFSA Journal*. (2019). 17(2):e05598.

10. European Food Safety Authority, Prevention ECfD, Control. The European Union Summary Report on Antimicrobial Resistance in zoonotic and indicator bacteria from humans, animals and food in 2017/2018. *EFSA Journal*. (2020). 18(3):e06007.

11. EFS Authority. The European Union Summary Report on Antimicrobial Resistance in zoonotic and indicator bacteria from humans, animals and food in 2018/2019. *EFSA Journal*. (2021). 19(4).

12. DC Bean, Livermore DM, Papa I, Hall LM. Resistance among Escherichia coli to sulphonamides and other antimicrobials now little used in man. *Journal of Antimicrobial Chemotherapy*. (2005). 56(5):962-4.

13. European Centre for Disease Prevention and Control. *Salmonellosis - Annual Epidemiological Report for 2017*. Stockholm: ECDC. (2020). Available.

14. A Cassini, Colzani E, Pini A, Mangen M-JJ, Plass D, McDonald SA, et al. Impact of infectious diseases on population health using incidence-based disability-adjusted life years (DALYs): results from the Burden of Communicable Diseases in Europe study, European Union and European Economic Area countries, 2009 to 2013. *Eurosurveillance*. (2018). 23(16):17-00454.

15. Eurostat. *Population and population change statistics.* European Commission. (2021) [updated 05/07/2021; cited 2022 02/02/2022]. Available from: <https://ec.europa.eu/eurostat/statistics-explained/index.php?title=Population_and_population_change_statistics#EU_population_shows_a_slight_decrease_in_2020>.

16. T Toni, Welch D, Strelkowa N, Ipsen A, Stumpf MP. Approximate Bayesian computation scheme for parameter inference and model selection in dynamical systems. *J R Soc Interface*. (2009). 6(31):187-202.

17. S Nair, Farzan A, O'Sullivan TL, Friendship RM. Time course of Salmonella shedding and antibody response in naturally infected pigs during grower-finisher stage. *Can J Vet Res*. (2018). 82(2):139-45.

18. World Health Organisation. *Salmonella (non-typhoidal).* Geneva: World Health Organisation. (2018) [Available from: <https://www.who.int/news-room/fact-sheets/detail/salmonella-(non-typhoidal>).

19. J Sheridan, Allen P, Ziegler J, Marinkov M, Suvakov M, Heinz G. Guidelines for slaughtering, meat cutting and further processing: FAO; 1991.

20. M Roser. *Life expectancy.* (2013) [Available from: <https://ourworldindata.org/life-expectancy>.

21. RE Kass, Raftery AE. Bayes factors. *Journal of the american statistical association*. (1995). 90(430):773-95.

22. A Saltelli, Bolado R. An alternative way to compute Fourier amplitude sensitivity test (FAST). *Computational Statistics & Data Analysis*. (1998). 26(4):445-60.

23. K Soetaert, Herman PM. A practical guide to ecological modelling: using R as a simulation platform: Springer; 2009.

24. AJ Bertrand Iooss, Gilles Pujol, with contributions from Khalid Boumhaout, Sebastien Da Veiga, Thibault Delage, Jana Fruth, Laurent Gilquin, Joseph Guillaume, Loic Le Gratiet, Paul Lemaitre, Barry L. Nelson, Filippo Monari, Roelof Oomen, Oldrich Rakovec, Bernardo Ramos, Olivier Roustant, Eunhye Song, Jeremy Staum, Roman Sueur, Taieb Touati and Frank Weber sensitivity: Global Sensitivity Analysis of Model Outputs. version 1.15.2 ed2018.

25. MBG Stefan Wilhelm. tmvtnorm: Truncated Multivariate Normal and Student t Distribution. 1.4-10 ed2015.

26. D Makowski, Ben-Shachar MS, Lüdecke D. bayestestR: Describing effects and their uncertainty, existence and significance within the Bayesian framework. *Journal of Open Source Software*. (2019). 4(40):1541.

27. H Wickham. Elegant graphics for data analysis. *Media*. (2009). 35(211):10.1007.

28. E Campitelli. metR: Tools for easier analysis of meteorological fields. 2020.

29. A Kassambara. Package ‘ggpubr’. 0.4.0 ed2020. p. abou

.

30. AA Baptiste Auguie. gridExtra: Miscellaneous Functions for "Grid" Graphics. 2.3 ed2017.
